# Supplementary figures and images for: Host phospholipid peroxidation fuels ExoU-dependent cell necrosis and supports Pseudomonas aeruginosa-driven pathology
Source: PLoS Pathog. 2021 Sep 13;17(9):e1009927. doi: 10.1371/journal.ppat.1009927 (PMC8460005; doi:10.1371/journal.ppat.1009927)

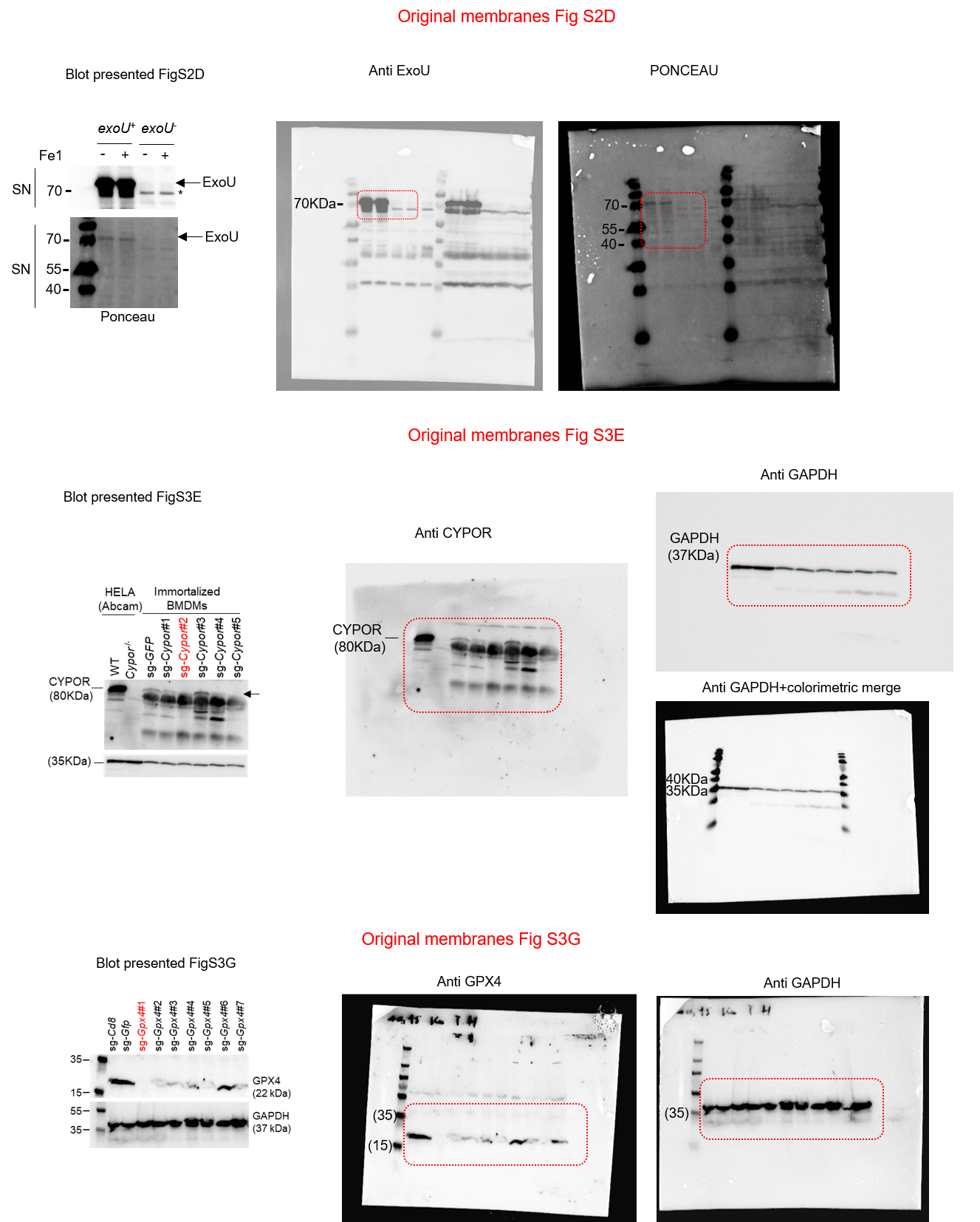

Supplement: S1 Data — (TIF) [file ppat.1009927.s001.tif]

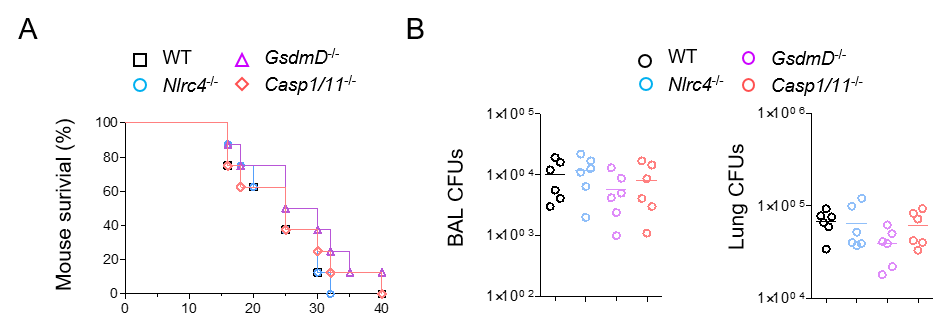

Supplement: S1 Fig — (A) Survival of WT, Casp1-/-/Casp11-/-, Nlrc4-/- and GsdmD-/- mice intranasally infected (n = 6 animals per condition) with 5.105 CFUs of P. aeruginosa PP34. Graphs represent one experiment (6 mice/group) out of three independent in vivo experiments. NS: Not significant using Log-rank Cox-Mantel test for survival comparisons. (B) Bronchoalveolar (BAL) and lung bacterial loads from WT, Casp1-/-/Casp11-/-, Nlrc4-/- and GsdmD-/- mice (n = 6) 18 hours after intranasal infection with 5.105 CFUs of P. aeruginosa PP34. Graphs represent one experiment (6 mice/group) out of three independent in vivo experiments. NS: Not significant using Mann-Whitney analysis test. (TIF) [file ppat.1009927.s003.tif]

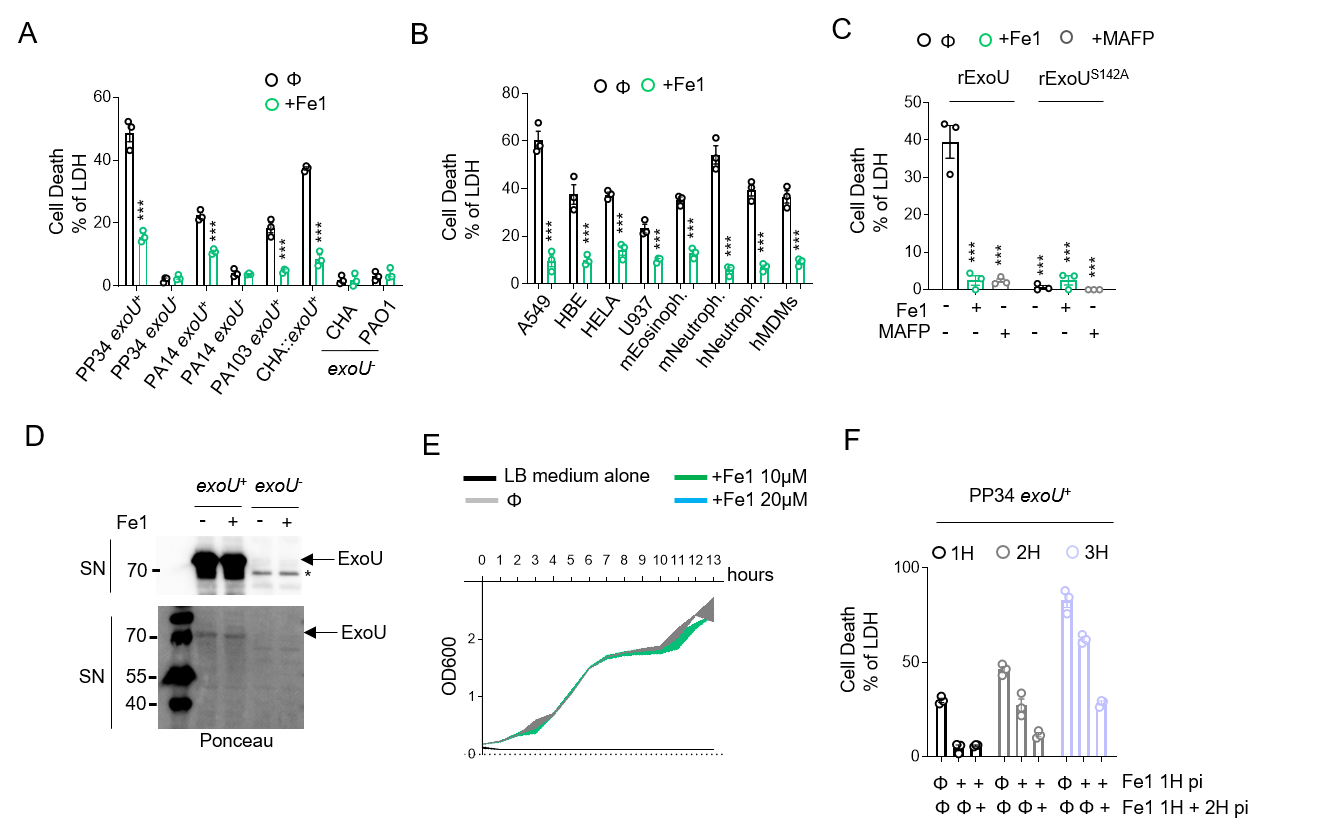

Supplement: S2 Fig — (A, B) Measure of LDH release in various human and murine cell types infected with various P. aeruginosa strains expressing or not exoU in presence of Ferrostatin-1 (Fe1, 10μM) for 2 hours. (C) LDH release in BMDMs transfected with recombinant ExoU (100ng) or its catalytically inactive mutant ExoUS142A, in presence of MAFP (50μM) or Ferrostatin-1 (Fe1, 10μM) for 3 hours. ***p ≤ 0.001, T-test with Bonferroni correction. (D) Immunoblotting of ExoU secretion by P. aeruginosa in presence of ferrostatin-1 (20μM). Star (*) show non-specific bands. (E) Measure of bacterial growth (O.D 600) in presence or absence of ferrostatin-1 (10, 20μM) for 14 hours). (F) Measure of LDH release in Nlrc4-/- BMDMs infected with PP34 (MOI5) in presence of Ferrostatin-1 (Fe1, 10μM) for 3 hours. Each hour, fresh Ferrostatin-1 (10μM) was added to cells (+) or not (φ). “pi” refers to post-infection. (TIF) [file ppat.1009927.s004.tif]

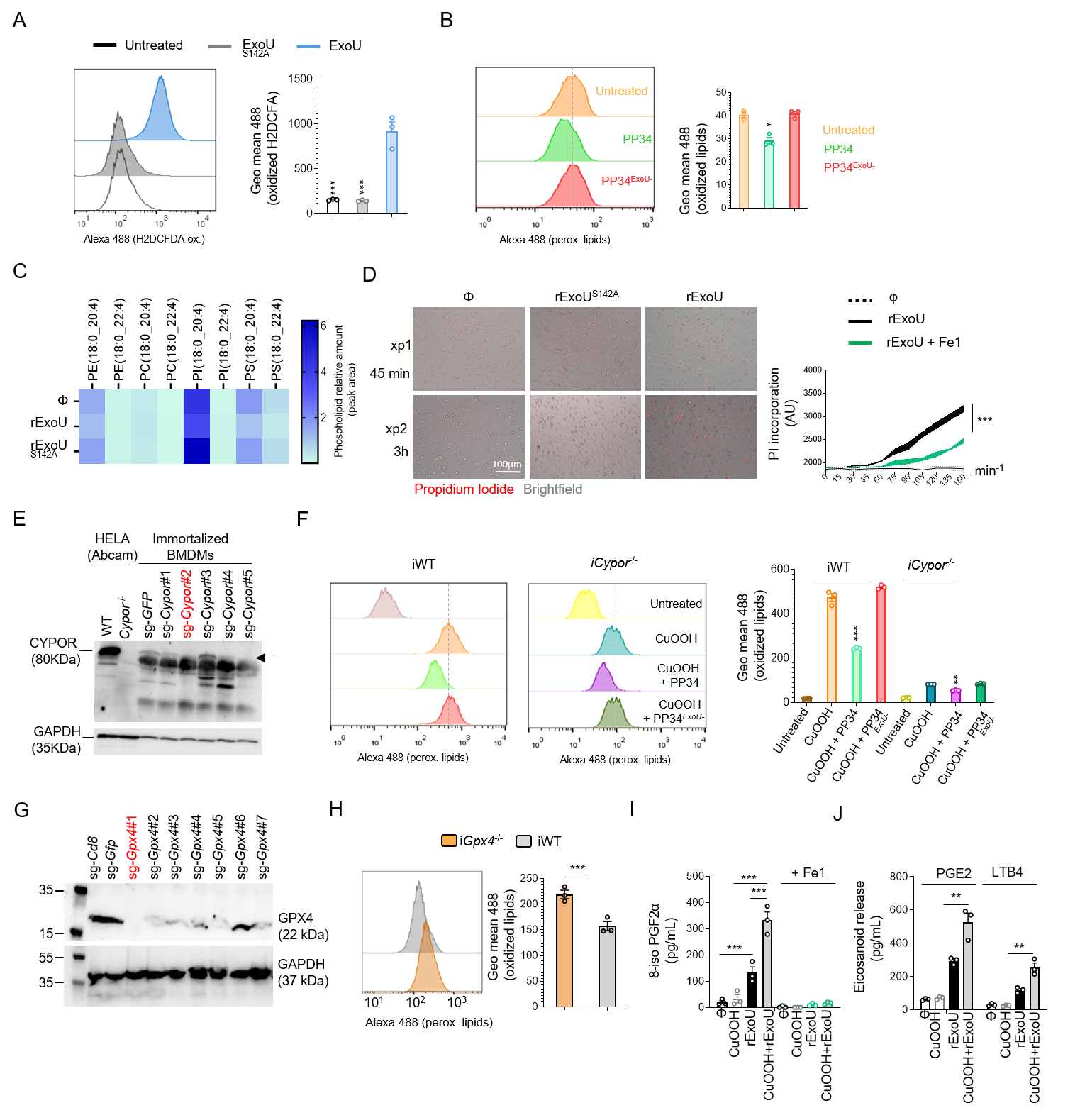

Supplement: S3 Fig — (A) ROS production in WT BMDMs transfected with ExoU or its catalytically dead mutant ExoUS142A for 45 minutes using H2DCFDA (1μM) probe. (B) Cytometry detection and quantification of (phospho)lipid peroxidation using the probe C11-bodipy in WT BMDMs infected with PP34ExoU+ or PP34ExoU- (MOI 5) for 1 hour. Sample were acquired using FACSCalibur (BD). The graph shows the mean+/-SEM of one experiment performed in triplicate out of three independent experiments. *P≤0.05, for the indicated comparisons using t-test with Bonferroni correction. (C) Lipidomic analysis of the relative amount of each phospholipid upon rExoU transfection analysed in Fig 3B. (D) Representative microscopy images and time course experiment of propidium iodide uptake in WT BMDMs transfected with rExoU or its catalytically inactive mutant ExoUS142A (500ng) in presence or not of ferrostatin-1 (Fe1, 10μM). Images show two independent experiments, each performed three times at 45 minutes or 3 hours post transfection. (E) Immunoblotting of Crispr Cas9-mediated Cypor gene deletion in immortalized (i)BMDMs or of Cypor-deficient HELA cells. The Cypor#2 (red) was selected for further analysis. GFP means that cells were transduced with sgRNA targeting Gfp and used as control. (F) Cytometry detection and quantification of (phospho)lipid peroxidation using the probe C11-bodipy in WT or Cypor-/- immortalized (i)BMDMs pre-treated or not for 1 hour with CuOOH (20μM) in presence or absence of Ferrostatin-1 (20μM) and then infected with PP34ExoU+ or PP34ExoU- (MOI 5) for 1 hour. Sample were acquired using FACSCalibur (BD). The graphs shows the mean+/-SEM of one experiment performed in triplicate out of three independent experiments. *P ≤ 0.05, **P≤0.001, for the indicated comparisons using t-test with Bonferroni correction. (G) Immunoblotting of Crispr Cas9-mediated Gpx4 gene deletion in immortalized BMDMs. The Gpx4#1 (red) was selected for further analysis. CD8 and GFP means that cells were transduced with sgRNA [file ppat.1009927.s005.tif]

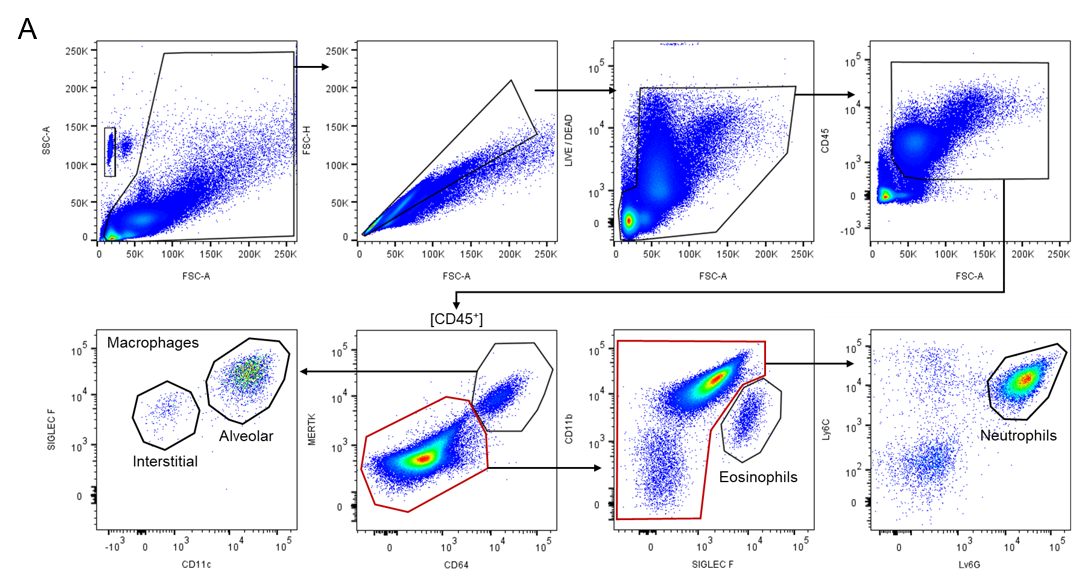

Supplement: S4 Fig — (A) Gating strategy to analyse Immune cell populations in bronchoalveolar fluids (BALFs). Immune cells were identified as CD45+ cells. Among CD45+ cells, different subset of immune cells including Interstitial/Alveolar Macrophages, Eosinophils and Neutrophils are identified based on specific cell surface marker expression. (TIF) [file ppat.1009927.s006.tif]

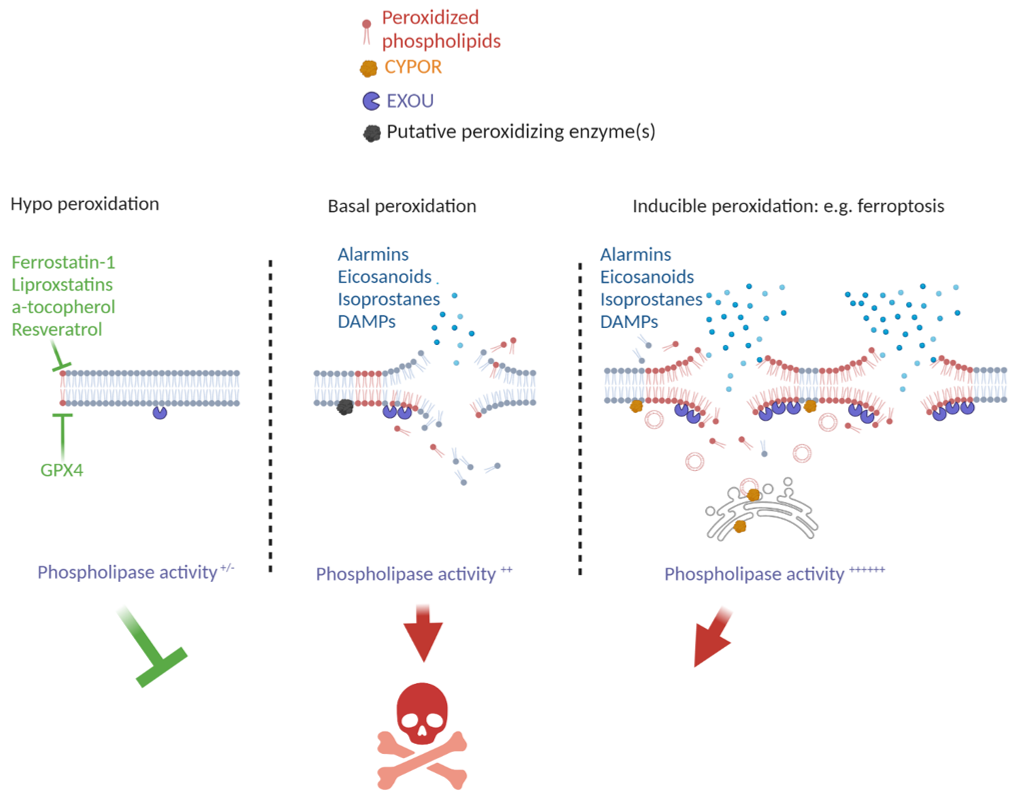

Supplement: S1 Graphical Abstract — In resting cells or in cells with induced lipid peroxidation (e.g. ferroptosis pathway), ExoU (purple) becomes hyper-activated by host cell peroxidised phospholipids, which drives an exacerbated cell necrosis, alarmin and lipid release and contributes to the subsequent pathology. Consequently, targeting lipid peroxidation (ferrostatin-1) inhibits ExoU-dependent cell necrosis and attenuates the host deleterious consequences. EM and SB used Biorender.com to create this figure. (TIF) [file ppat.1009927.s007.tif]
