# Supplementary material for: Host phospholipid peroxidation fuels ExoU-dependent cell necrosis and supports Pseudomonas aeruginosa-driven pathology
Source: PLoS Pathog. 2021 Sep 13;17(9):e1009927. doi: 10.1371/journal.ppat.1009927 (PMC8460005; doi:10.1371/journal.ppat.1009927)
Supplement: Attachment — Submitted filename: Response to reviewers.pdf [file ppat.1009927.s020.pdf]

We thank the reviewers and the editor for their insightful comments and suggestions that help to strengthen the manuscript. We have accordingly answer to all comments either experimentally or by discussing as proposed by the referee. Overall, we are confident that they improved both the manuscript and the scientific findings.

Reviewer's Responses to Questions

PartI-Summary

Please use this section to discuss strengths/weaknesses of study, novelty/significance, general execution and scholarship.

Reviewer #1: The research article entitled, “Host phospholipid peroxidation fuels ExoU-dependent cell necrosis and supports Pseudomonas aeruginosa-driven pathology” is an interesting and important study that further clarifies the mechanism by which ExoU causes host cell lysis in a variety of cell types. The authors present strong evidence to support the hypothesis that ExoU injected into the cytosol of host cells by bacterial type III secretion system utilizes the natural process of membrane lipid peroxidation to enhance ExoU phospholipase A2 activity leading to rapid necrosis. Oxidized lipids serve as signals for the cellular process of ferroptosis and increasing or inhibiting ferroptosis through modulation of lipid peroxidation appears to modulate ExoU activity in parallel. Two main conclusions remain somewhat unclear from these studies;

We thank reviewer 1 for highlighting the novelty of ExoU at using host-peroxidized phospholipids to mediate its pathological cell necrosis.

(1) How does exoU mediated necrosis deviate from ferroptosis? If lipid peroxidation enhances both ExoU-mediated necrosis and feroptosis, is there a means to prevent ferroptosis is the context of increased lipid peroxidation that would preserve ExoU-mediated necrosis or vice versa? Are other triggers of necrosis resistant to feostatin-1 treatment when they do not depend on lipid peroxidation? In a balanced lipid peroxidative state is would seem that the presence of ExoU exploits the basal state of lipid peroxidation to cleave at the sn2 position and this heightened PLA2 activity may serve to destabilize the membrane, however, in a heightened lipid peroxidative state where the cell is destined for ferroptosis, does ExoU necrosis override this process and what are distinguishing consequences, if any, between a cell undergoing ExoU-mediated exocytosis or ferroptosis? Is alarmin release an exclusive indicator of cell necrosis or does this occur during ferroptosis? In Figure 2D, is there a reason why there is no phenotype in alox12/15-/- cells? Is 15LOX-1 in mice critical means of oxidizing membrane phospholipids?

"How does exoU mediated necrosis deviate from ferroptosis? If lipid peroxidation enhances both ExoU-mediated necrosis and feroptosis, is there a means to prevent ferroptosis is the context of increased lipid peroxidation that would preserve ExoU-mediated necrosis or vice versa?",

We further investigated experimentally the interactions between ferroptosis pathway and ExoU. As mentioned in our manuscript, we have used Cumen hydroperoxide (CuOOH), a lipid peroxidation agent but also a ferroptosis inducer when used at high concentrations (300-500µM). To answer the first question regarding the ability of ExoU to exploit ferroptosis pathway, we used cells in which we invalidated the major enzyme involved in CuOOH-dependent phospholipid peroxidation, namely cytochrome oxidioreducatse p450 (CYPOR). In WT cells CuOOH induce lipid peroxidation, a process strongly impaired in CYPOR-/- cells (Figure B, Figure S3-F of the revised version). Therefore, the infection of CuOOH-primed cells increased ExoU-dependent cell necrosis. To the contrary, such increase was completely lost in CYPOR-/- cells, suggesting that upon inducible lipid peroxidation, ExoU-triggered exacerbated cell necrosis benefits from ferroptosis-inducible pathways. Cytometry experiments measuring lipid peroxidation showed that ExoU also decreased CYPOR-dependent lipid peroxidation, hence suggesting that indeed, upon ferroptosis induction program (CYPOR-dependent lipid peroxidation), ExoU-exploits this pathway to promote phospholipase-dependent cellular necrosis.

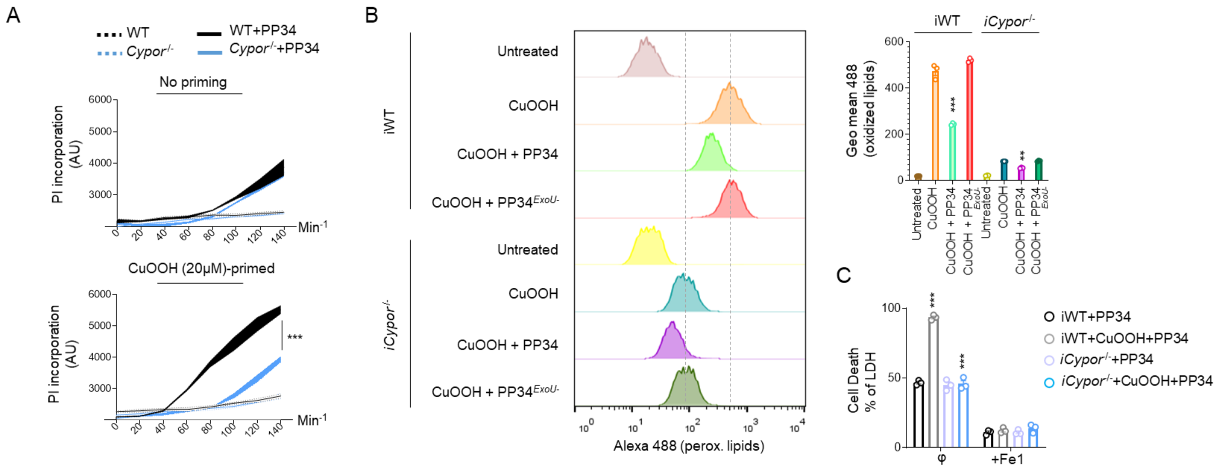

A. Time course measure of plasma membrane permeabilization using propidium iodide incorporation in WT and Cypor-/- HeaA cells primed or not with CuOOH (20µM, 1hour) and infected with PP34 (MOI5) for 2 hours. ***p ≤ 0.001, T-test with Bonferroni correction.

B. Cytometry detection and quantification of (phospho)lipid peroxidation using the probe C11-bodipy in WT or Cypor-/- immortalized (i)BMDMs pre-treated or not for 1 hour with CuOOH (20µM) in presence or absence of Ferrostatin-1 (20µM) and then infected with PP34ExoU+ or PP34ExoU- (MOI 5) for 1 hour. Sample were acquired using FACSCalibur™ (BD). The graphs shows the mean+/-SEM of one experiment performed in triplicate out of three independent experiments. *P ≤ 0.05, **P≤0.001, for the indicated comparisons using t-test with Bonferroni correction.

C. Measure of LDH release in immortalized (i) WT or Cypor-/- BMDMs primed or not with CuOOH (20µM, 1hour) in presence or absence of ferrostatin-1 (20µM) and infected for 2 hours with PP34. ***p ≤ 0.001, T-test with Bonferroni correction.

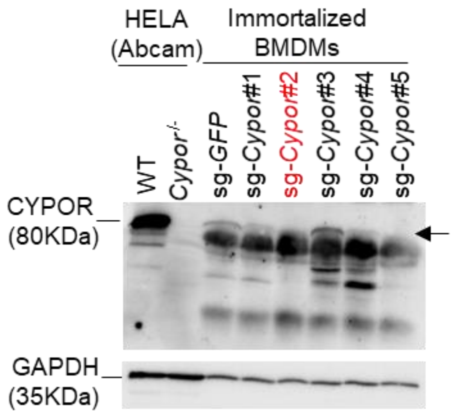

Immunoblotting of Crispr Cas9-mediated Cypor gene deletion in immortalized (i)BMDMs or of Cypor-deficient HeLa cells. The Cypor#2 (red) was selected for further analysis. GFP means that cells were transduced with sgRNA targeting Gfp and used as control. Arrow show the 80kDa size corresponding to Human and murine CYPOR Molecular Weight.

"Are other triggers of necrosis resistant to feostatin-1 treatment when they do not depend on lipid peroxidation?"

We evaluated the importance of Ferrostatin-1 against pyroptosis and necroptosis: None of those cell necrosis relied on lipid peroxidation to undergo lysis (measured as the % of LDH release). Such observations have also been observed by Wiernicki et al., regarding the importance of lipid peroxidation in pyroptosis and necroptosis (Wiernicki et al., 2020). Therefore, such process seems to be specific to ExoU- and ExoU-related virulence factors. This has been included in Figure 2 of the manuscript.

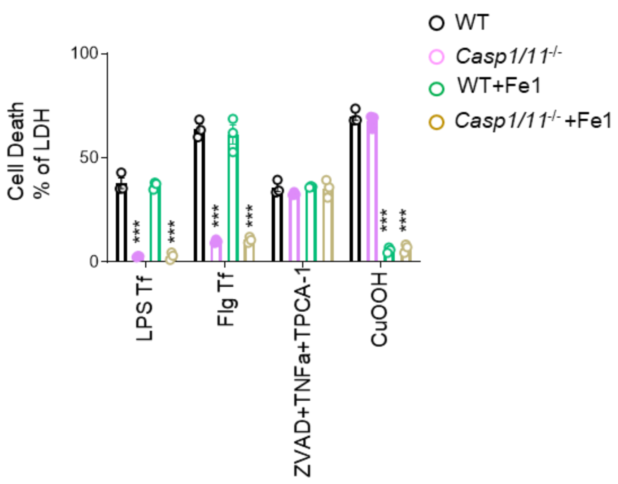

Measure of LDH release in WT or Casp1-/-/Casp11-/- BMDMs transfected (Lipofectamin2000) with 1µg of LPS or Flagellin (Flg) to induce pyroptosis, treated with Z-VAD (40µM)/TNFα (500UI/mL)/TPCA-1 (5µM) to induce necroptosis or with Cumene hydroperoxide (CuOOH, 400µM) to induce ferroptosis in presence or absence of Ferrostatin-1 (Fe1, 10µM) for 6 hours. ***$p \leq 0.001$, T-test with Bonferroni correction.

"Is alarmin release an exclusive indicator of cell necrosis or does this occur during ferroptosis?"

This is a very intriguing point. Indeed, ferroptosis has been described for 9 years now, and only few reports have tried to decipher if it can promote alarmin release like pyroptosis, necroptosis, NETosis or secondary/late/lytic apoptosis. So far, various studies showed that HMGB1, an alarmin released by cellular necrosis, is released upon ferroptosis, suggesting that to some extent, ferroptosis might be close to other regulated cell necrosis (Wen et al., 2019). Regarding ferroptosis, and its specific signature, further investigations will be required to determine if 1/ classical alarmins/DAMPs associate with such cell death and 2/ if there are specific alarmins/DAMPs released by ferroptotic cells.

We compared alarmin release (e.g. IL1a/HMGB1) in BMDMs infected with PP34 ExoU or stimulated to undergo ferroptosis (CuOOOH 500µM). The results below show that both ferroptosis and PP34 ExoU induce release of IL1a and HMGB1, hence suggesting that alarmin release is also a hallmark of ferrotposis. Although our work has shown some interconnections with ferroptosis, we respectfully wish not to include these results in our current manuscript as

it mostly address fundamental ferroptosis questions that go beyond the ExoU work but also because we currently work on the specific signature of alarmin and DAMP generated in various ferroptotic contexts.

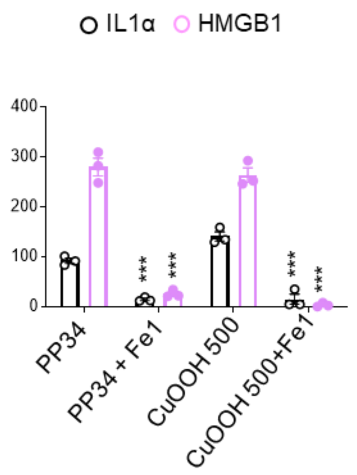

Measure of alarmin release in Pam3CSK4 (TLR2-ligand)-primed WT BMDMs infected with PP34ExoU+ (MOI2) or treated with Cumene hydroperoxide (CuOOH, 500µM) to induce ferroptosis in presence or absence of Ferrostatin-1 (Fe1, 10µM) for 6 hours. ***$p \leq 0.001$, T-test with Bonferroni correction

"In Figure 2D, is there a reason why there is no phenotype in alox12/15-/- cells? Is 15LOX-1 in mice critical means of oxidizing membrane phospholipids?"

The role of ALOX12/15 or human 15-1 LOX in phospholipid peroxidation-dependent ferroptosis remains currently extensively debated (Jiang et al., 2021). Indeed, upon ferroptosis induction by the erastin molecule, siRNA targeting all human LOXs show that there is a reduction in ferroptosis induction (Yang et al., 2016). However, the use of various KO human or murine models failed to show an involvement of ALOX12/15, or human 12LOX or 15 LOX at regulating lipid peroxidation and ferroptosis upon exposure to other ferrotposis inducers such as RSL3, FIN56, ML210, CuOOH or even erastin in some studies (Jiang et al., 2021). In addition, the use of various lipoxygenase inhibitors have showed a strong radical trapping activity (RTA) in addition to their lipoxygenase inhibiting properties (Shah et al., 2018). Specifically, the RTA from LOX inhibitors, more than their enzymatic activity has been shown to be responsible of ferroptosis inhibition observed in various experiments (Shah et al., 2018). Therefore, it seems that LOXs might have a phenotype in certain contexts, which in our settings does not seem to occur. To the contrary, CuOOH-/RSL3-induced lipid peroxidation occurs in a CYPOR-dependent manner ((Ai et al., 2021), (Zou et al., 2020a) and our study). There are many pathways that can promote lipid peroxidation either at a basal state or in ferroptosis-inducible contexts, such as CYPOR, LOXs, Squalen oxidase, a oxidase, nadph oxidase or iron-mediated non-enzymatic lipid peroxidation (Jiang et al., 2021). Hence, we speculate that in

resting cells, all those enzymes and non-enzymatic processes might play complementary and redundantly roles at promoting basal lipid peroxidation. Such area is currently under deep investigations in the ferroptosis field, but still does not have a clear answer.

To suppress from here:

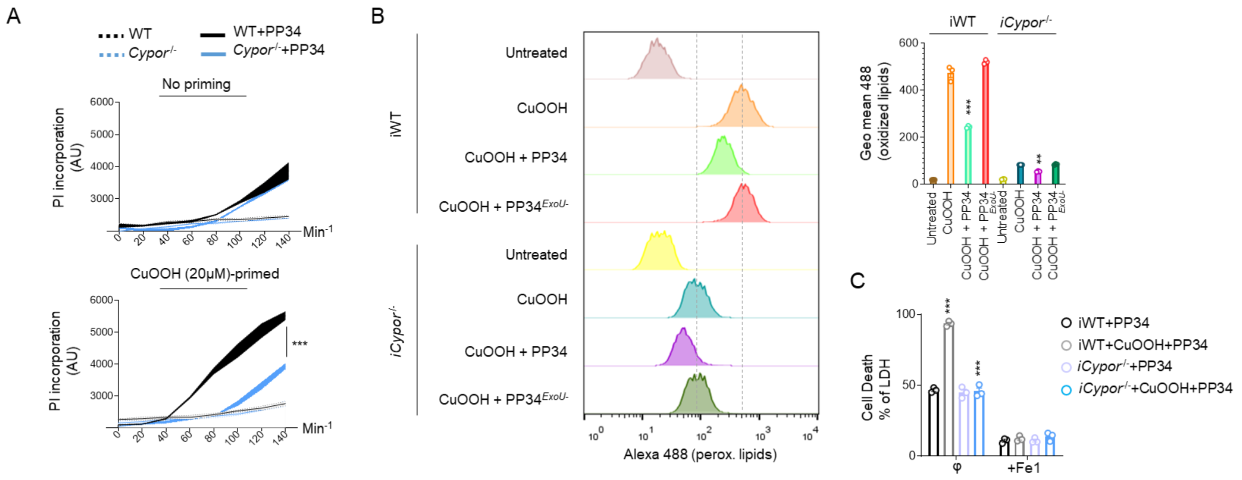

A. Time course measure of plasma membrane permeabilization using propidium iodide incorporation in WT and Cypor-/- HeLa cells primed or not with CuOOH (20µM, 1hour) and infected with PP34 (MOI5) for 2 hours. ***p ≤ 0.001, T-test with Bonferroni correction.

B. Cytometry detection and quantification of (phospho)lipid peroxidation using the probe C11-bodipy in WT or Cypor-/- immortalized (i)BMDMs pre-treated or not for 1 hour with CuOOH (20µM) in presence or absence of Ferrostatin-1 (20µM) and then infected with PP34ExoU+ or PP34ExoU- (MOI 5) for 1 hour. Sample were acquired using FACSCalibur™ (BD). The graphs shows the mean+/-SEM of one experiment performed in triplicate out of three independent experiments. *P ≤ 0.05, **P≤0.001, for the indicated comparisons using t-test with Bonferroni correction.

C. Measure of LDH release in immortalized (i) WT or Cypor-/- BMDMs primed or not with CuOOH (20µM, 1hour) in presence or absence of ferrostatin-1 (20µM) and infected for 2 hours with PP34. ***p ≤ 0.001, T-test with Bonferroni correction.

(2) For in vivo studies, what is the relative contribution of cellular necrosis versus elaboration of eicosanoid inflammatory mediators and recruitment of immune cells (which precedes necrosis) toward the lung pathology? What cell types in vivo are the most consequential target of ExoU that result in pathology. Since ferrostatin-1 inhibits lipid peroxidation, it would be predicted by the conclusion, "ExoU-targeted peroxidised phospholipids might increase its phospholipase activity toward all phospholipids (peroxidized or not)", that both cellular necrosis and enhanced release of inflammatory mediator would be interfered with by ferrostatin-1 treatment. The authors draw the conclusion that, "Although a pathological function of recruited immune cells such as neutrophils is probable, we hypothesize that ferrostatin-1-inhibited resident alveolar macrophage death in response to exoU-expressing P. aeruginosa might confer an improved immune protection characterized by lower immune cell recruitment and lower tissue damages". It could also be hypothesized that ferrostatin-1 reduces the magnitude of eicosanoid generation for mediators such as LTB4, which result in less neutrophil recruitment and less immune cell-mediated damage. Further studies to experimentally disconnect necrosis from enhanced eicosanoid generation by ExoU would be necessary to delineate the relative role in pathology. Such insight would better resolve in vivo mechanisms to inform the development of treatment strategies. These issues warrant being addressed within the Discussion.

We thank reviewer 1 for these very insightful comments and suggestions. Indeed, at this step, we cannot rule out that ferrostatin-1-inhibited ExoU-cleaved phospholipids might help mice surviving *Pseudomonas* infection by down regulating LTB4 production and subsequent neutrophil recruitment. An easy guess for us would be that both LTB4/other eicosanoids and cellular necrosis contribute to ExoU-dependent pathology.

A central experiment would be to infect ALOX5 deficient mice, that lack LTB4 production capacity, a process that mostly occurs in neutrophils, and to a lower extend in macrophages. Our in vitro experiments show that alox 5 does not modulate macrophage cell necrosis upon ExoU intoxication. However, we apologize, as we could not perform in vivo infections of ALOX5 +/- ferrostatin-1 due to the strong reduction of the current mouse colony in our collaborator lab that arises from the current pandemic situation. Therefore, we discuss this aspect in the discussion as being an important point to elucidate in future studies. Accordingly we included in the discussion section the following sentences: "Regarding this, our *in vivo* observations that targeting lipid peroxidation confers some protection of mice against ExoU-dependent pathology is to put in the light of a decrease in some eicosanoid production such as LTB4 and PGE2, two important modulators of ExoU-driven pathology (Machado et al., 2011;

Sadikot et al., 2007; Saliba et al., 2005). Therefore, the use of Alox5-/- or Cox-/- mice, unable to generate LTB4 or various prostaglandins respectively, will also help to determine the respective importance of eicosanoid burst or cell necrosis upon lipid peroxidation-driven ExoU activity."

The graphic abstract could be significantly improved to better clarify the story of ExoU impact by including time sequence as well as balanced state, hyper and hypo-lipid peroxisation states. We fully agree. In this context, the graphical abstract has been substantially modified and various peroxidized phospholipid states/situations have been included in order to clarify and improve the graphical abstract message.

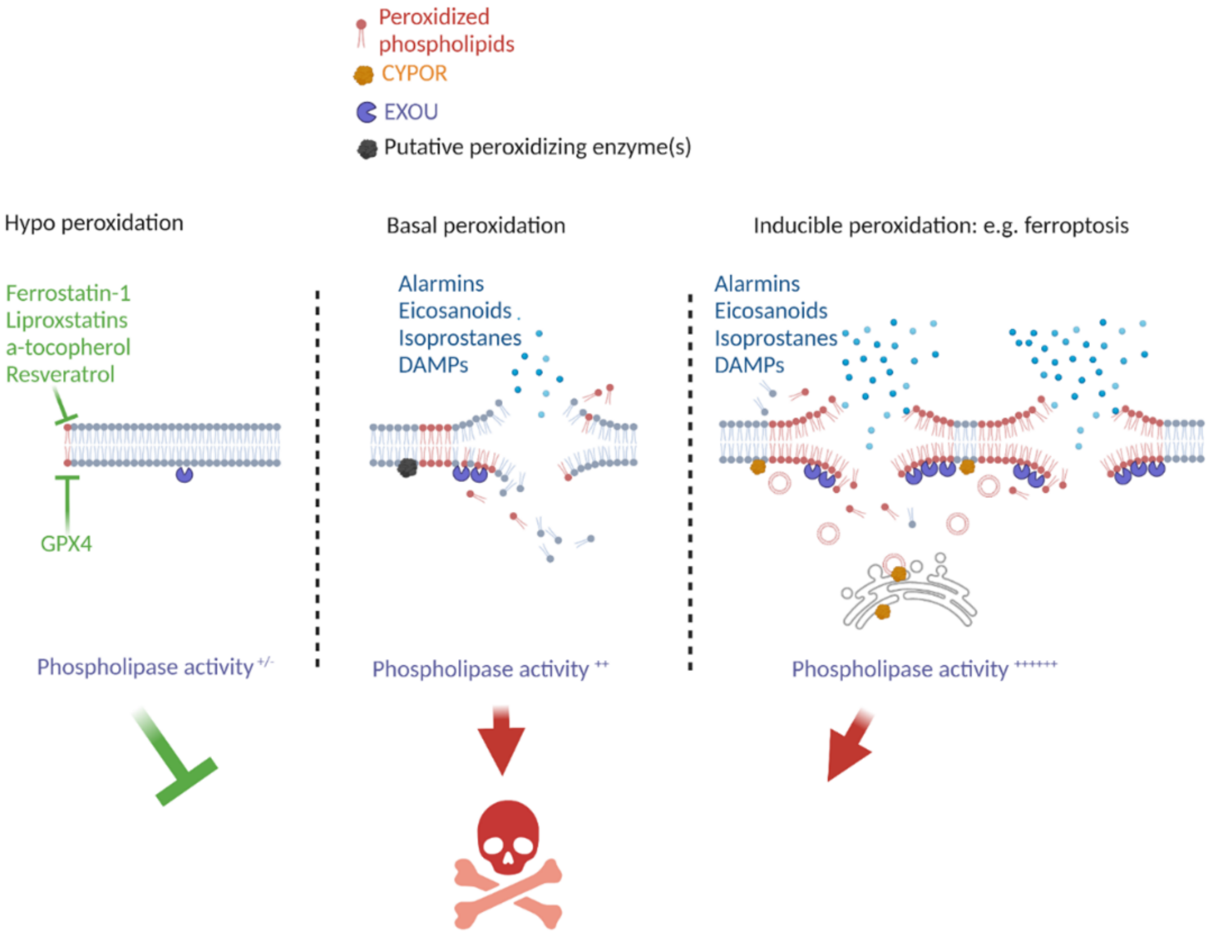

Graphical abstract: Host lipid peroxidation fuels ExoU-induced cell necrosis-dependent pathology. In resting cells or in cells with induced lipid peroxidation (e.g. CYPOR-induced ferroptosis pathway), ExoU (purple) becomes hyper-activated by host cell peroxidised phospholipids, which drives an exacerbated cell necrosis, alarmin and lipid release and contributes to the subsequent pathology. Consequently, targeting lipid peroxidation (ferrostatin-1) inhibits ExoU-dependent cell necrosis and attenuates the host deleterious consequences. Created with Biorender.com

Reviewer #2: Bakgayoko et al., reports a fundamental aspect of host-pathogen interactions, namely the interference with signaling pathways of their host cell by intracellular pathogens. This is a carefully written and experimentally well conducted study that demonstrates the exploitation of host phospholipids by P. aeruginosa. Here the authors demonstrate, a phospholipase from P. aeruginosa, ExoU, triggers host lipid peroxidation and induces ROS mediated necrosis in the host cell. ExoU mediated necrosis is counter effected by Ferrostatin-1 further validates the hypothesis. Overall, this is an interesting study in the context of understanding the P. aeruginosa induced pathology. If the concerns listed below can be addressed, I recommend this manuscript for publication.

We thank reviewer 2 for those encouraging and positive comments. We have accordingly tried to address them in the following parts of the rebuttal.

Reviewer #3: The manuscript presents a novel point of view about the exploitation of host oxidative mechanisms by Pseudomonas aeruginosa. According to the authors. P. aeruginosa uses the endogenous basal lipid peroxidation by the virulence factor ExoU to promote cell death. The study was well conducted and shows scientific relevance.

We thank reviewer 3 for underlining the interest and relevance of our study. Accordingly, we have addressed the suggested comments in the rebuttal part.

Part II – Major Issues: Key Experiments Required for Acceptance

Please use this section to detail the key new experiments or modifications of existing experiments that should be absolutely required to validate study conclusions.

Generally, there should be no more than 3 such required experiments or major modifications for a "Major Revision" recommendation. If more than 3 experiments are necessary to validate the study conclusions, then you are encouraged to recommend "Reject".

Reviewer #1: In aggregate this is a well-designed study with important implications. The biggest weakness lies in effectively articulating the findings. The graphic abstract is insufficient and there are some confusing statements and inconsistencies leaving the reader a little confused despite compelling data. The role of ferroptosis in all this and how it interplays with cellular necrosis is not addressed. Does ExoU interfere with ferroptosis in order to drive necrosis or is ExoU functioning at a basal non-ferroptosis state?

If you trigger ferroptosis, thereby enhancing ExoU-driven necrosis, does the ferroptosis process have any consequence to the cell or is necrosis the dominant process in that context?

“How does exoU mediated necrosis deviate from ferroptosis? If lipid peroxidation enhances both ExoU-mediated necrosis and feroptosis, is there a means to prevent ferroptosis is the context of increased lipid peroxidation that would preserve ExoU-mediated necrosis or vice versa?”,

We further investigated experimentally the interactions between ferroptosis pathway and ExoU. As mentioned in our manuscript, we have used Cumen hydroperoxide (CuOOH), a lipid peroxidation agent but also a ferroptosis inducer when used at high concentrations (300-500µM). To answer the first question regarding the ability of ExoU to exploit ferroptosis pathway, we used cells in which we invalidated the major enzyme involved in CuOOH-dependent phospholipid peroxidation, namely cytochrome oxidioreducatse p450 (CYPOR). In WT cells CuOOH induce lipid peroxidation, a process strongly impaired in CYPOR-/- cells. Therefore, the infection of CuOOH-primed cells increased ExoU-dependent cell necrosis. To the contrary, such increase was completely lost in CYPOR-/- cells, suggesting that upon inducible lipid peroxidation, ExoU-triggered exacerbated cell necrosis benefits from ferroptosis-inducible pathways. Cytometry experiments measuring lipid peroxidation showed that ExoU also decreased CYPOR-dependent lipid peroxidation, hence suggesting that indeed, upon ferroptosis induction program (CYPOR-dependent lipid peroxidation), ExoU-exploits this pathway to promote phospholipase-dependent cellular necrosis.

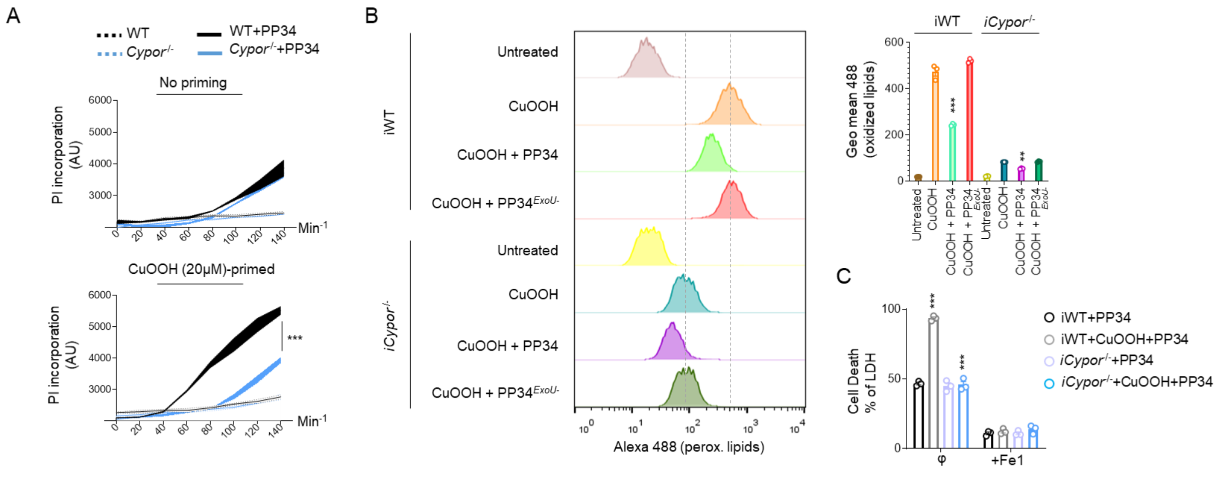

A. Time course measure of plasma membrane permeabilization using propidium iodide incorporation in WT and Cypor-/- HeLa cells primed or not with CuOOH (20µM, 1hour) and infected with PP34 (MOI5) for 2 hours. ***p ≤ 0.001, T-test with Bonferroni correction.

B. Cytometry detection and quantification of (phospho)lipid peroxidation using the probe C11-bodipy in WT or Cypor-/- immortalized (i)BMDMs pre-treated or not for 1 hour with CuOOH (20µM) in presence or absence of Ferrostatin-1 (20µM) and then infected with PP34ExoU+ or PP34ExoU- (MOI 5) for 1 hour. Sample were acquired using FACSCalibur™ (BD). The graphs shows the mean+/-SEM of one experiment performed in triplicate out of

three independent experiments. *P ≤ 0.05, **P≤0.001, for the indicated comparisons using t-test with Bonferroni correction.

C. Measure of LDH release in immortalized (i) WT or Cypor-/- BMDMs primed or not with CuOOH (20µM, 1hour) in presence or absence of ferrostatin-1 (20µM) and infected for 2 hours with PP34. ***p ≤ 0.001, T-test with Bonferroni correction.

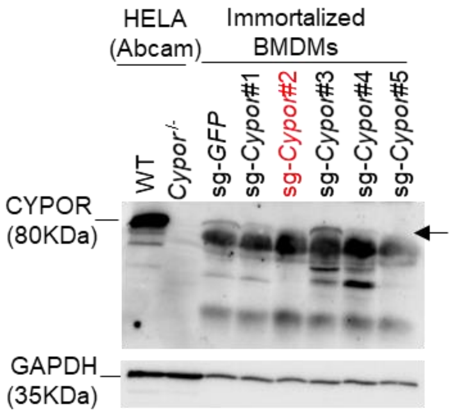

Immunoblotting of Crispr Cas9-mediated Cypor gene deletion in immortalized (i)BMDMs or of Cypor-deficient HeLa cells. The Cypor#2 (red) was selected for further analysis. GFP means that cells were transduced with sgRNA targeting Gfp and used as control. Arrow show the 80kDa size corresponding to Human and murine CYPOR Molecular Weight.

"Are other triggers of necrosis resistant to feostatin-1 treatment when they do not depend on lipid peroxidation?"

We evaluated the importance of Ferrostatin-1 against pyroptosis and necroptosis: None of those cell necrosis relied on lipid peroxidation to undergo lysis (measured as the % of LDH release). Such observations have also been observed by Wiernicki et al., regarding the importance of lipid peroxidation in pyroptosis and necroptosis (Wiernicki et al., 2020). Therefore, such process seems to be specific to ExoU- and ExoU-related virulence factors. This has been included in Figure 2 of the manuscript.

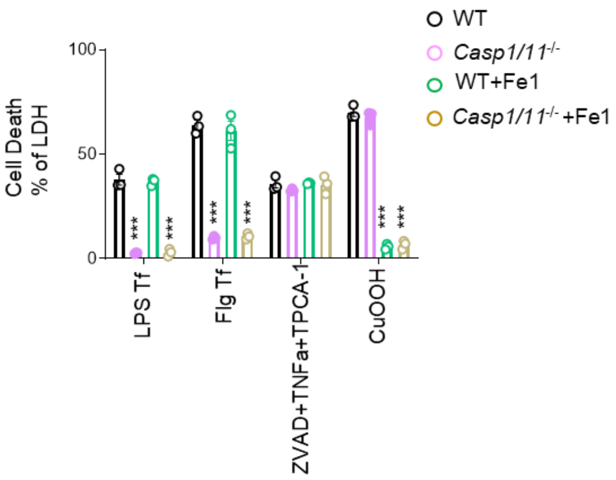

Measure of LDH release in WT or Casp1-/-/Casp11-/- BMDMs transfected (Lipofectamin2000) with 1µg of LPS or Flagellin (Flg) to induce pyroptosis, treated with ZVAD (40µM)/TNFα (500UI/mL)/TPCA-1 (5µM) to induce necroptosis or with Cumene hydroperoxide (CuOOH, 400µM) to induce ferroptosis in presence or absence of Ferrostatin-1 (Fe1, 10µM) for 6 hours. ***p ≤ 0.001, T-test with Bonferroni correction.

"Is alarmin release an exclusive indicator of cell necrosis or does this occur during ferroptosis?"

This is a very intriguing point. Indeed, ferroptosis has been described for 9 years now, and only few reports have tried to decipher if it ca promote alarmin release like pyroptosis, necroptosis, NETosis or secondary/late/lytic apoptosis. So far, various studies showed that HMGB1, an alarmin released by cellular necrosis, is released upon ferroptosis, suggesting that to some extent, ferroptosis might be close to other regulated cell necrosis (Wen et al., 2019). Regarding ferroptosis, and its specific signature, further investigations will be required to determine if 1/ classical alarmins/DAMPs associate with such cell death and 2/ if there are specific alarmins/DAMPs released by ferroptotic cells.

We compared alarmin release (e.g. IL1a/HMGB1) in BMDMs infected with PP34ExoU or stimulated to undergo ferroptosis (CuOOOH 500µM). The results below show that both ferroptosis and PP34ExoU induce release of IL1a and HMGB1, hence suggesting that alarmin release is also a hallmark of ferrotposis. Although our work has shown some interconnections with ferroptosis, we respectfully wish not to include these results in our current manuscript as it mostly address fundamental ferroptosis questions that go beyond the ExoU work but also because we currently work on the specific signature of alarmin and DAMP generated in various ferroptotic contexts.

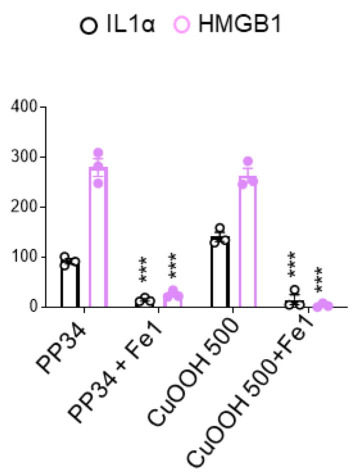

Measure of alarmin release in Pam3CSK4 (TLR2-ligand)-primed WT BMDMs infected with PP34ExoU+ (MOI2) or treated with Cumene hydroperoxide (CuOOH, 500µM) to induce ferroptosis in presence or absence of Ferrostatin-1 (Fe1, 10µM) for 6 hours. ***$p \leq 0.001$, T-test with Bonferroni correction

"In Figure 2D, is there a reason why there is no phenotype in alox12/15-/- cells? Is 15LOX-1 in mice critical means of oxidizing membrane phospholipids?"

The role of ALOX12/15 or human 15-1 LOX in phospholipid peroxidation-dependent ferroptosis remains currently extensively debated (Jiang et al., 2021). Indeed, upon ferroptosis induction by the erastin molecule, siRNA targeting all human LOXs show that there is a reduction in ferroptosis induction (Yang et al., 2016). However, the use of various KO human or murine models failed to show an involvement of ALOX12/15, or human 12LOX or 15 LOX at regulating lipid peroxidation and ferroptosis upon exposure to other ferrotposis inducers such as RSL3, FIN56, ML210, CuOOH or even erastin in some studies (Jiang et al., 2021). In addition, the use of various lipoxygenase inhibitors have showed a strong radical trapping activity (RTA) in addition to their lipoxygenase inhibiting properties (Shah et al., 2018). Specifically, the RTA from LOX inhibitors, more than their enzymatic activity has been shown to be responsible of ferroptosis inhibition observed in various experiments (Shah et al., 2018). Therefore, it seems that LOXs might have a phenotype in certain contexts, which in our settings does not seem to occur. To the contrary, CuOOH-/RSL3-induced lipid peroxidation occurs in a CYPOR-dependent manner ((Ai et al., 2021), (Zou et al., 2020a) and our study). There are many pathways that can promote lipid peroxidation either at a basal state or in ferroptosis-inducible contexts, such as CYPOR, LOXs, Squalen oxidase, a oxidase, nadph oxidase or iron-mediated non-enzymatic lipid peroxidation (Jiang et al., 2021). Hence, we speculate that in resting cells, all those enzymes and non-enzymatic processes might play complementary and redundantly roles at promoting basal lipid peroxidation. Such area is currently under deep investigations in the ferroptosis field, but still does not have a clear answer.

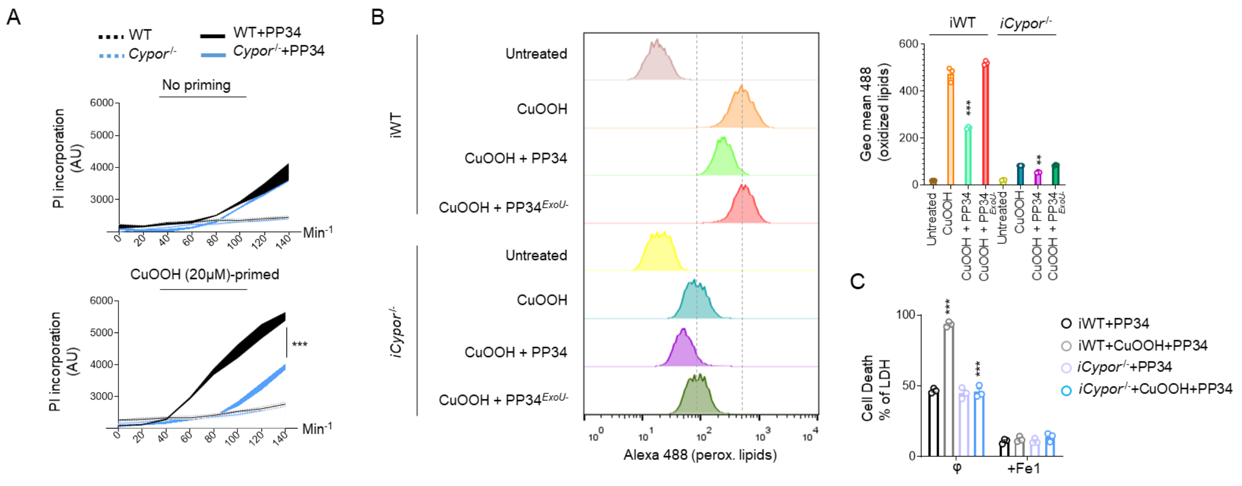

A. Time course measure of plasma membrane permeabilization using propidium iodide incorporation in WT and

Cypor-/- HeLa cells primed or not with CuOOH (20µM, 1hour) and infected with PP34 (MOI5) for 2 hours. ***p ≤ 0.001, T-test with Bonferroni correction.

B. Cytometry detection and quantification of (phospho)lipid peroxidation using the probe C11-bodipy in WT or Cypor-/- immortalized (i)BMDMs pre-treated or not for 1 hour with CuOOH (20µM) in presence or absence of Ferrostatin-1 (20µM) and then infected with PP34ExoU+ or PP34ExoU- (MOI 5) for 1 hour. Sample were acquired using FACSCalibur™ (BD). The graphs shows the mean+/-SEM of one experiment performed in triplicate out of three independent experiments. *P ≤ 0.05, **P≤0.001, for the indicated comparisons using t-test with Bonferroni correction.

C. Measure of LDH release in immortalized (i) WT or Cypor-/- BMDMs primed or not with CuOOH (20µM, 1hour) in presence or absence of ferrostatin-1 (20µM) and infected for 2 hours with PP34. ***p ≤ 0.001, T-test with Bonferroni correction.

(2) For in vivo studies, what is the relative contribution of cellular necrosis versus elaboration of eicosanoid inflammatory mediators and recruitment of immune cells (which precedes necrosis) toward the lung pathology? What cell types in vivo are the most consequential target of ExoU that result in pathology. Since ferrostatin-1 inhibits lipid peroxidation, it would be predicted by the conclusion, "ExoU-targeted peroxidised phospholipids might increase its phospholipase activity toward all phospholipids (peroxidized or not)", that both cellular necrosis and enhanced release of inflammatory mediator would be interfered with by ferrostatin-1 treatment. The authors draw the conclusion that, "Although a pathological function of recruited immune cells such as neutrophils is probable, we hypothesize that ferrostatin-1-inhibited resident alveolar macrophage death in response to exoU-expressing P. aeruginosa might confer an improved immune protection characterized by lower immune cell recruitment and lower tissue damages". It could also be hypothesized that ferrostatin-1 reduces the magnitude of eicosanoid generation for mediators such as LTB4, which result in less neutrophil recruitment and less immune cell-mediated damage. Further studies to experimentally disconnect necrosis from enhanced eicosanoid generation by ExoU would be necessary to delineate the relative role in pathology. Such insight would better resolve in vivo mechanisms to inform the development of treatment strategies. These issues warrant being addressed within the Discussion.

We thank reviewer 1 for these very insightful comments and suggestions. Indeed, at this step, we cannot rule out that ferrostatin-1-inhibited ExoU-cleaved phospholipids might help mice surviving *Pseudomonas* infection by down regulating LTB4 production and subsequent neutrophil recruitment. An easy guess for us would be that both LTB4/other eicosanoids and cellular necrosis contribute to ExoU-dependent pathology.

A central experiment would be to infect ALOX5 deficient mice, that lack LTB4 production capacity, a process that mostly occurs in neutrophils, and to a lower extend in macrophages. Our in vitro experiments show that alox 5 does not modulate macrophage cell necrosis upon ExoU intoxication. However, we apologize, as we could not perform in vivo infections of ALOX5 +/- ferrostatin-1 due to the strong reduction of the current mouse colony in our collaborator lab that arises from the current pandemic situation. Therefore, we discuss this aspect in the discussion as being an important point to elucidate in future studies. Accordingly we included in the discussion section the following sentences: "Regarding this, our *in vivo* observations that targeting lipid peroxidation confers some protection of mice against ExoU-dependent pathology is to put in the light of a decrease in some eicosanoid production such as

LTB4 and PGE2, two important modulators of ExoU-driven pathology (Machado et al., 2011; Sadikot et al., 2007; Saliba et al., 2005). Therefore, the use of Alox5-/- or Cox-/- mice, unable to generate LTB4 or various prostaglandins respectively, will also help to determine the respective importance of eicosanoid burst or cell necrosis upon lipid peroxidation-driven ExoU activity."

The graphic abstract could be significantly improved to better clarify the story of ExoU impact by including time sequence as well as balanced state, hyper and hypo-lipid peroxisation states. We fully agree. In this context, the graphical abstract has been substantially modified and various peroxidized phospholipid states/situations have been included in order to clarify and improve the graphical abstract message.

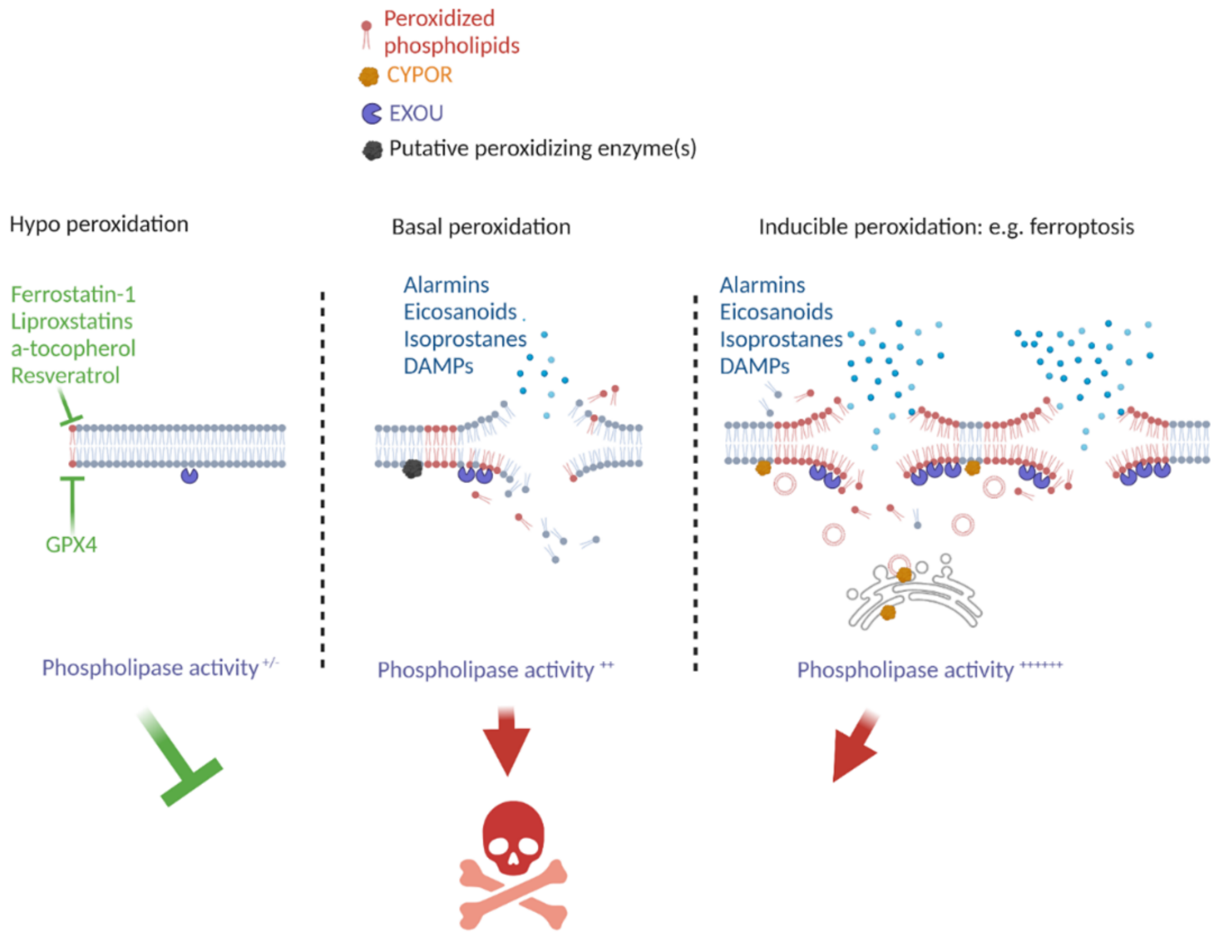

Graphical abstract: Host lipid peroxidation fuels ExoU-induced cell necrosis-dependent pathology. In resting cells or in cells with induced lipid peroxidation (e.g. CYPOR-induced ferroptosis pathway), ExoU (purple) becomes hyper-activated by host cell peroxidised phospholipids, which drives an exacerbated cell necrosis, alarmin and lipid release and contributes to the subsequent pathology. Consequently, targeting lipid peroxidation (ferrostatin-1) inhibits ExoU-dependent cell necrosis and attenuates the host deleterious consequences. Created with Biorender.com

Reviewer #2: Major points:

1) Explain how did the authors validate successful ExoU mutation?

Validation of successful mutations were done by our collaborators that generated those strains and proteins. They have previously validated the lack of activity of those constructs/strains using various biochemical and biological settings such as (Deruelle et al., 2020). The reference has been integrated in the material and method section in order to validate the efficacy of those tools/strains.

In addition, our phospholipase assays show that the catalytically dead mutant ExoUS142 is not able to promote phospholipid cleavage in cell lysates, a process that still occur with WT ExoU (**Figure 3I**).

2) I strongly feel Figure S1 which demonstrates the effect is inflammasome independent is important result in the context of this study and suggest the data should be shown in the main text rather than in supplement.

We thank our reviewer for this suggestion. Indeed, we agree that this is an important aspect of the work. However, a study from (Al Moussawi and Kazmierczak, 2014) also showed that Caspase-1/11 dKO mice are not better protected from ExoU-expressing P. aeruginosa infection, which somehow lowers the importance of this figure. Therefore, we would like to keep it as supplemental, as we believe that these experiments, despite being important, mostly synthesis what has been published previously by (Al Moussawi and Kazmierczak, 2014).

3) What are the cellular sources of ROS production? And the source of the lipids that are susceptible for ExoU induced peroxidation. Probably, H2DCFDA and Bodipy stained immunofluorescence assay with organelle markers can address this. I understand these experiments are time consuming and if the authors feel it is out of scope of the current study, I suggest discussing couple of lines on this, based on what is known already.

We thank reviewer 2 for these suggestions. Indeed, this is a central question explored by various groups currently. The Dixon lab has suggested using bodipy probes that plasma membrane carries the peroxidized phospholipids responsible of ferroptosis (Magtanong et al., 2019). In addition, the Kagan and Conrad labs showed that phosphatydil ethanolamines (PEs) are the crucial phospholipids responsible of ferroptosis induction (Aldrovandi et al., 2018; Doll et al., 2017; Kagan et al., 2017). However, many other phospholipids are found to be peroxidized upon ferrotposis, in various cellular compartments, including cardiolipins in mictochondria,

PS/PC/PI in ER/plasma membrane, which renders difficult determining their importance in ferroptosis induction (Abstract, 2019; Aldrovandi et al., 2018; Doll et al., 2017; Kagan et al., 2017; Zou et al., 2020b). In our settings, ExoU does not drive lipid peroxidation, but degrades lipid peroxidation to trigger cell necrosis. In this context, we found that peroxidzed PS and PC are decreased upon ExoU exposure (**Figure 3 in manuscript**), suggesting that one or both phospholipids might be heavily targeted by ExoU. We are currently investigating the role(s) for various PS/PC acyl transferases in the frame of exoU toxicity, but have not yet generated significant results on it. Therefore, we have tried to determine if, as for ferrotposis, ExoU could exploit peroxidized phospholipids at the plasma membrane and in the endoplasmic reticulum. Indeed, ExoU activity has been shown to be important at the plasma membrane and the ER is a central provider of phospholipids to the plasmame membrane by contributing to the Land Cycle (Kagan et al., 2017; Magtanong et al., 2019; Zou et al., 2020b). In microscopy, it is very difficult to follow peroxidzed lipids in resting cells by using fluorescent microscopy. Therefore, to improve our signal sensitivity, we infected with ExoU-expressing PP34 HeLa cells primed with 20µM of Cumene hydroperoxide (CuOOH) to induced phospholipid peroxidation. Using the bodipy probe, we found accumuluation of peroxidized phospholipids at the plasma membrane as well as in the ER compartment. In addition, infection with PP34 promoted a robust decrease in peroxidized phospholipids from the plasma membrane, suggesting to us that although ER might contribute to provide peroxidized phospholipids, ExoU mostly targets peroxidized phospholipids at the plasma membrane. Accordingly, those results were integrated in the main text but also discussed regarding the current scientific knowledge in the discussion part as follows: "<u>Although controlled phospholipid peroxidation is of importance for the cells to perform various processes such as efferocytosis through the engagement of peroxidised-PS, mitochondria-dependent apoptosis through cardiolipin peroxidation, signal transduction through peroxidised PC-derived lipids, unrestricted accumulation of peroxidised PEs drives ferroptosis (Bochkov et al., 2010; Tyurin et al., 2014; Tyurina et al., 2019). A question in both basal lipid peroxidation and ferroptosis-induced lipid peroxidation lies on the compartment phospholipid peroxidation occurs. Peroxisiomes are key at providing ether-phospholipids that will be peroxidised upon ferroptosis induction, the Endoplasmic reticulum is also a central regulator of phospholipid turn over and plasma membrane constitutes the probable location of lipid peroxidation-driven cell lysis upon ferroptosis induction (Kagan et al., 2017; Magtanong et al., 2019; Zou et al., 2020b). Our observations also suggest that although lipid peroxidation can occur in various cellular compartments, ExoU-induced cell necrosis mostly arises from plasma membrane cleaved peroxidized phospholipids. Yet, this does not exclude at all that</u>

phospholipid peroxidation could occur in other intracellular organelles, including the endoplasmic reticulum."

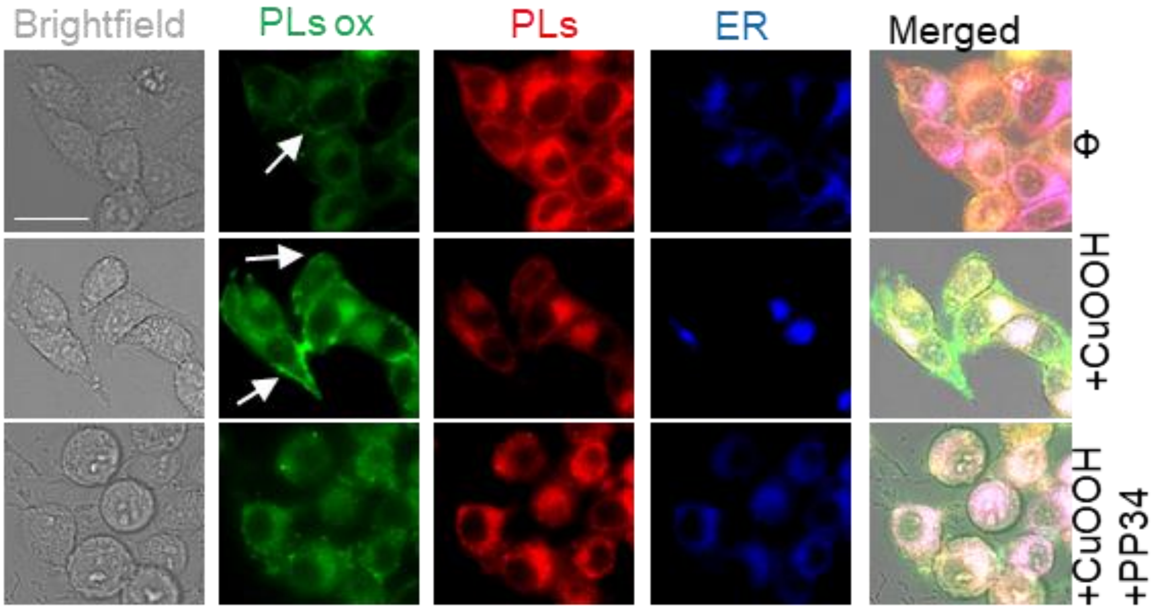

Representative microscopy images (phospho)lipid peroxidation using the probe C11-bodipy in CuOOH-primed (20µM) HeLa cells infected with PP34 (MOI5) for 2 hours. Images show two independent experiments, each performed three times at 2 hours post infection. Scale bar 20µm; Green, oxidized bodipy (oxidized phospholipids, PLs); Red, bodipy (phospholipids, PLs); Blue (Endoplasmic Reticulum, ER). Arrows show enriched peroxidsed phospholipids in the plasma membrane area.

Regarding the ROS source, we do not have a clear answer yet. We have used NADPH oxidase KO cells (Gp91phoxKO), NOS2KO, Gp91phox/NOS2dKO, ALOX12/15KO, ALOX5 KO, CYPOR KO and found no phenotype in those cells at basal state. As low ROS levels are sufficient to trigger basal lipid peroxidation and given that many enzymatic and non-enzymatic processes occur in cells, we speculate that most of those processes work in a redundantly or complementary manner to perform basal lipid peroxidation in cells. We are currently trying to identify the cellular source(s) of ROS, therefore we ask not to include those results in the current manuscript.

Regarding the inducible lipid peroxidation, we found that cumen hydroperoxide (CuOOH) that we used in our experiments to induce lipid peroxidation, is driven by the endoplasmic reticulum/microsomal enzyme CYPOR (cytochrome p450 oxidoreducatse), and that this helps ExoU to trigger cell necrosis in inducible lipid peroxidation contexts (please see Figure A below).

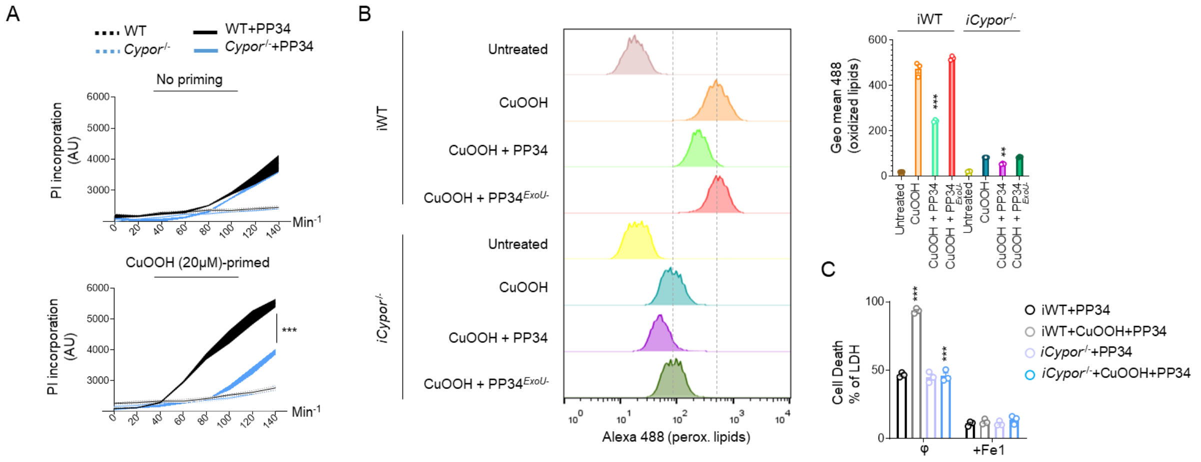

A. Time course measure of plasma membrane permeabilization using propidium iodide incorporation in WT and Cypor-/- HeLa cells primed or not with CuOOH (20µM, 1hour) and infected with PP34 (MOI5) for 2 hours. ***p ≤ 0.001, T-test with Bonferroni correction.

B. Cytometry detection and quantification of (phospho)lipid peroxidation using the probe C11-bodipy in WT or Cypor-/- immortalized (i)BMDMs pre-treated or not for 1 hour with CuOOH (20µM) in presence or absence of Ferrostatin-1 (20µM) and then infected with PP34ExoU+ or PP34ExoU- (MOI 5) for 1 hour. Sample were acquired using FACSCalibur™ (BD). The graphs shows the mean+/-SEM of one experiment performed in triplicate out of three independent experiments. *P ≤ 0.05, **P≤0.001, for the indicated comparisons using t-test with Bonferroni correction.

C. Measure of LDH release in immortalized (i) WT or Cypor-/- BMDMs primed or not with CuOOH (20µM, 1hour) in presence or absence of ferrostatin-1 (20µM) and infected for 2 hours with PP34. ***p ≤ 0.001, T-test with Bonferroni correction.

4) When the effect of ExoU is suppressed by Ferrostatin-1 and Liproxstatin-1, why does the authors still refer it as necrosis and rule out the possibility of ferroptosis. Explanation needed.

We thank reviewer 2 for pointing out this. Although we observe that either basal lipid peroxidation or induced lipid peroxidation are of importance to promote exacerbated ExoU-dependent cell necrosis, we cannot formally refer to this cell death as ferroptosis as we do not see an induction of phospholipid peroxidation upon ExoU intoxication but more a decrease in peroxidzed phospholipids, which suggests to us that ExoU, by cleaving or being activated

preferentially by peroxidzed phospholipids induces cellular necrosis (Jiang et al., 2021). Therefore, we think that ExoU phospholipase exploits the endogenous pathways that promote lipid peroxidation, including the deleterious one driven by ferroptosis triggers (CuOOH, cumene hydroperoxide). To support this, we found that induction of the ferroptosis pathways by stimulating CYPOR-dependent peroxidized phospholipid accumulation strongly exacerbated ExoU-dependent cell necrosis, a process that was not seen in Cypor-deficient cells, where basal lipid peroxidation (ferroptosis-independent) was important for ExoU-induced necrosis.

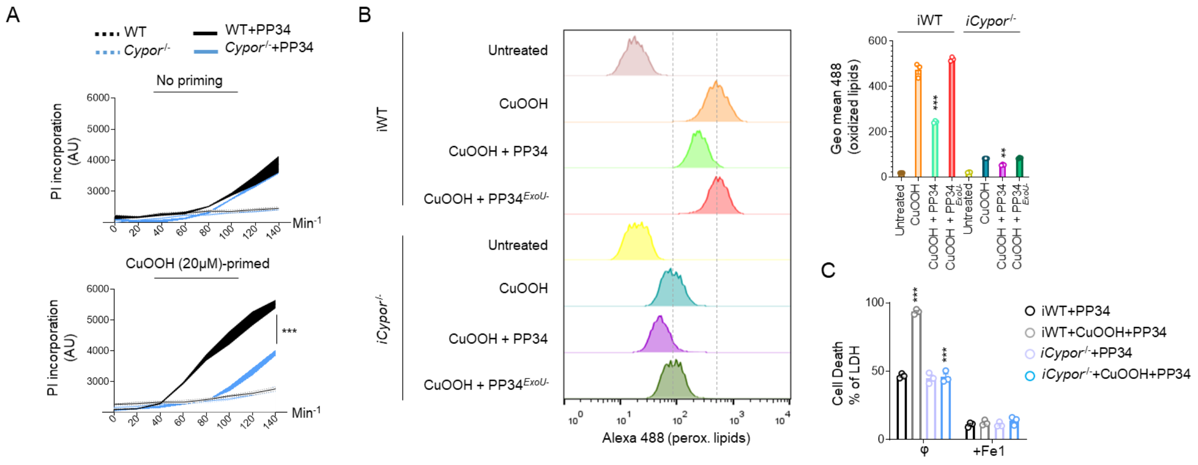

A. Time course measure of plasma membrane permeabilization using propidium iodide incorporation in WT and Cypor-/- HeaA cells primed or not with CuOOH (20µM, 1hour) and infected with PP34 (MOI5) for 2 hours. ***p ≤ 0.001, T-test with Bonferroni correction.

B. Cytometry detection and quantification of (phospho)lipid peroxidation using the probe C11-bodipy in WT or Cypor-/- immortalized (i)BMDMs pre-treated or not for 1 hour with CuOOH (20µM) in presence or absence of Ferrostatin-1 (20µM) and then infected with PP34ExoU+ or PP34ExoU- (MOI 5) for 1 hour. Sample were acquired using FACSCalibur™ (BD). The graphs shows the mean+/-SEM of one experiment performed in triplicate out of three independent experiments. *P ≤ 0.05, **P≤0.001, for the indicated comparisons using t-test with Bonferroni correction.

C. Measure of LDH release in immortalized (i) WT or Cypor-/- BMDMs primed or not with CuOOH (20µM, 1hour) in presence or absence of ferrostatin-1 (20µM) and infected for 2 hours with PP34. ***p ≤ 0.001, T-test with Bonferroni correction.

5) As rExoU failed to induce lipid peroxidation (Fig 3A), I doubt the oxidized lipids could be of bacterial origin which could result in increase in ROS in host cell and collapse. I strongly suggest having an IFA read out for C11-Bodipy to locate the source of lipid peroxidation.

Indeed, this is an important question to us as well. As mentioned earlier, unfortunately, we do not have a clear answer, but still are trying to tackle this. We have used NADPH oxidase KO cells, ALOX12/15 KO, ALOX5 KO and found no phenotype in those cells. As low ROS levels are sufficient to trigger basal lipid peroxidation and given that many enzymatic and non-

enzymatic processes occur in cells, we speculate that most of those processes work in a redundantly or complementary manner to perform basal lipid peroxidation in cells. Regarding the inducible lipid peroxidation, we found that cumen hydroperoxide (CuOOH) that we used in our experiments to induce lipid peroxidation, is driven by the endoplasmic reticulum/microsomal enzyme CYPOR (cytochrome p450 oxidoreducatse), and that this helps ExoU to trigger cell necrosis in inducible lipid peroxidation contexts.

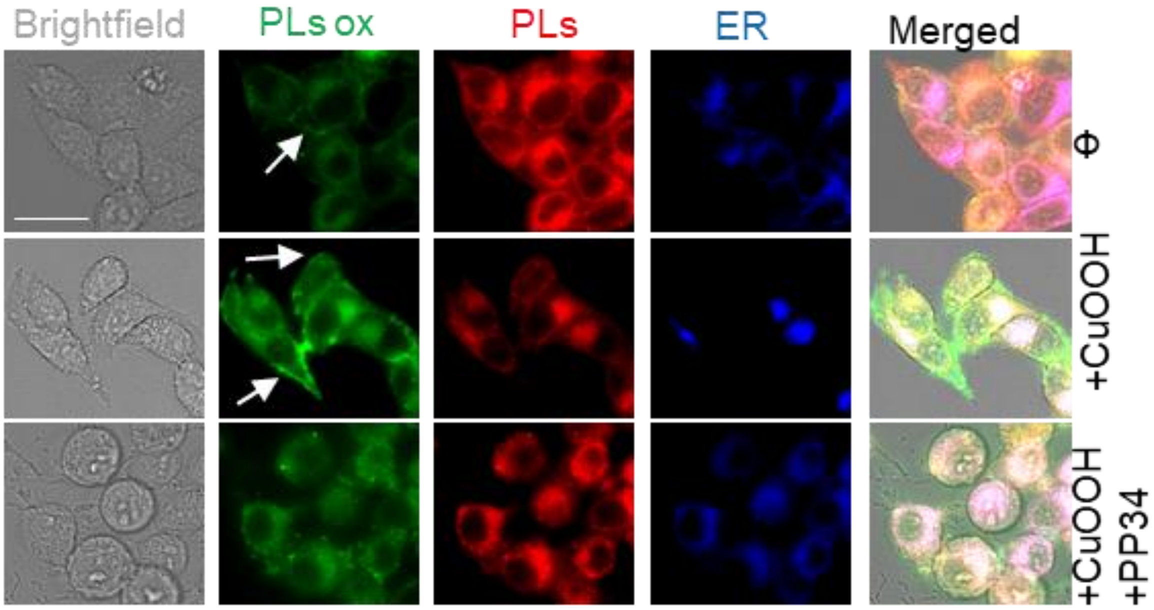

Representative microscopy images (phospho)lipid peroxidation using the probe C11-bodipy in CuOOH-primed (20µM) HeLa cells infected with PP34 (MOI5) for 2 hours. Images show two independent experiments, each performed three times at 2 hours post infection. Scale bar 20µm; Green, oxidized bodipy (oxidized phospholipids, PLs); Red, bodipy (phospholipids, PLs); Blue (Endoplasmic Reticulum, ER). Arrows show enriched peroxidsed phospholipids in the plasma membrane area.

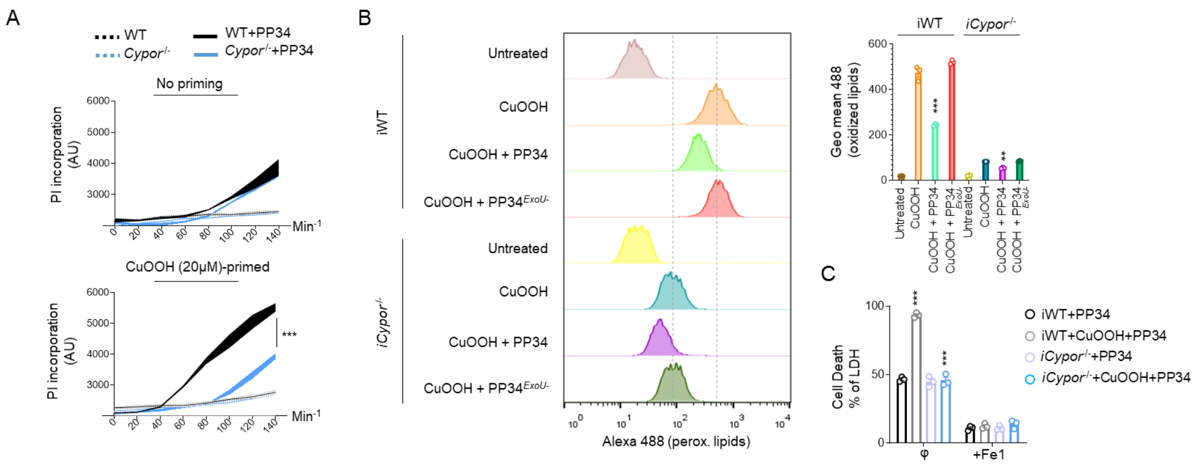

A. Time course measure of plasma membrane permeabilization using propidium iodide incorporation in WT and Cypor-/- HeLa cells primed or not with CuOOH (20µM, 1hour) and infected with PP34 (MOI5) for 2 hours. ***p ≤ 0.001, T-test with Bonferroni correction.

B. Cytometry detection and quantification of (phospho)lipid peroxidation using the probe C11-bodipy in WT or Cypor-/- immortalized (i)BMDMs pre-treated or not for 1 hour with CuOOH (20µM) in presence or absence of Ferrostatin-1 (20µM) and then infected with PP34ExoU+ or PP34ExoU- (MOI 5) for 1 hour. Sample were acquired using FACSCalibur™ (BD). The graphs shows the mean+/-SEM of one experiment performed in triplicate out of three independent experiments. *P ≤ 0.05, **P≤0.001, for the indicated comparisons using t-test with Bonferroni correction.

C. Measure of LDH release in immortalized (i) WT or Cypor-/- BMDMs primed or not with CuOOH (20µM, 1hour) in presence or absence of ferrostatin-1 (20µM) and infected for 2 hours with PP34. ***p ≤ 0.001, T-test with Bonferroni correction.

Reviewer #3: To improve the manuscript, I have the following suggestions:

1. The hypothesis is based on the findings presented in figure 3, which show a reduced level in oxidized lipids in cultures transfected with recombinant ExoU in comparison to the observed in non-transfected cultures.

To achieve these results, the authors performed the experiments using a 45 min time-point because at this time they did not detect any plasma membrane permeabilization by propidium iodide uptake (propidium uptake was monitored at 45 min and 3 hours). Therefore, the authors considered that, by using this time-point, they would exclude the involvemet of the ExoU-induced cell necrosis in the decrease of peroxidised phospholipids. However, Since Sato et al., (2003) have reported that ExoU is able to decrease the viability of yeast cells only 30 minutes after ExoU induction from a low copy number plasmid, the authors should show the exact time (between 45 min and 3 h) of the first signs of necrosis in their model.

We thank reviewer 3 for its suggestion. Accordingly, we performed a time course experiment to determine the critical time point of ExoU-induced cell necrosis. In our settings, we observed plasma membrane permeabilization on the time range of 45-60 minutes. We believe that the time where cell death will starts mostly depends on various parameters, such as ExoU transfection efficiency, amount of ExoU, the biology of cells, the type targeted cell… This result has been added in “FigS3D”.

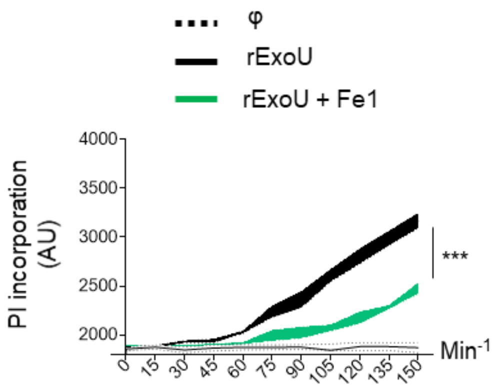

Time course experiment of propidium iodide uptake in WT BMDMs transfected with rExoU in presence or not of ferrostatin-1 (Fe1, 10µM).

2. The authors should show the levels of oxidized lipids in cultures infected with viable bacteria (ExoU+ and ExoU-) to strengthen the data obtained with recombinant ExoU.

In agreement with our reviewer suggestion, we performed infections with viable bacteria in presence or absence of Cumene hydroperoxide priming in cells and measured lipid peroxidation. Accordingly, we observed the same trends than using rExoU, hence confirming that viable bacteria are also able to exploit peroxided phospholipids, a process that requires ExoU expression. Those results have been included in Figure 3 and Figure S3.

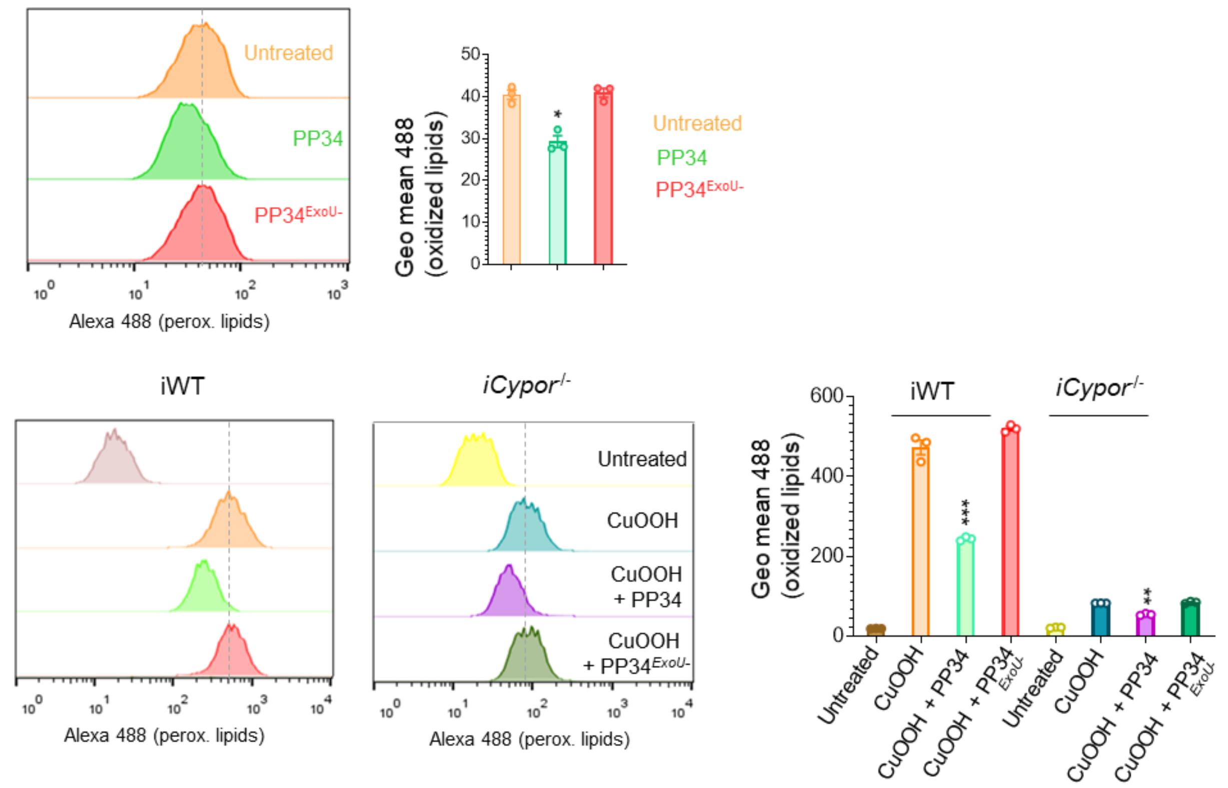

A. Cytometry detection and quantification of (phospho)lipid peroxidation using the probe C11-bodipy in (Moi je resterais sur les HeLa ou limite les C1C11 ? car dans les WT tu as de la pyro qui lyse les cellule est qui pourrait faire que ce résultat est un artifact lié à la mort) WT BMDMs infected with PP34ExoU+ or PP34ExoU- (MOI 5) for 1 hour. Sample were acquired using FACSCalibur™ (BD). The graph shows the mean+/-SEM of one experiment performed in triplicate out of three independent experiments. *P≤0.05, for the indicated comparisons using t-test with Bonferroni correction.

B. Cytometry detection and quantification of (phospho)lipid peroxidation using the probe C11-bodipy in WT or Cypor-/- immortalized (i)BMDMs pre-treated or not for 1 hour with CuOOH (20µM) in presence or absence of Ferrostatin-1 (20µM) and then infected with PP34ExoU+ or PP34ExoU- (MOI 5) for 1 hour. Sample were acquired using FACSCalibur™ (BD). The graphs shows the mean+/-SEM of one experiment performed in triplicate out of three independent experiments. *P ≤ 0.05, **P≤0.001, for the indicated comparisons using t-test with Bonferroni correction.

3. Based on Figure 2E, the authors reported that ferrostatin-1 delayed the ExoU-induced cell necrosis, but did not conclude whether this effect had resulted from the ferrostatin-1 instability over time or from a lipid peroxidation-independent cell death mediated by the phospholipase activity of ExoU. Since ferrostatin1 did not interfere in bacterial viability or ExoU secretion, the authors should add fresh medium containing ferrostatin-1 to solve this question.

We fully agree, therefore, we performed an experiment to determine if ferrostatin-1 instability could account for the lipid-peroxidation-independent action of ExoU. We replenished the medium with ferostatin-1 each hour and measured for 3hours the ExoU-induced necrosis levels (LDH release). Indeed, ferostatin-1 instability played a significant role in lipid peroxidation-independent action of ExoU as fresh ferrostatin-1 further improved resistance of cells to ExoU intoxication. This has been included in Figure S2.

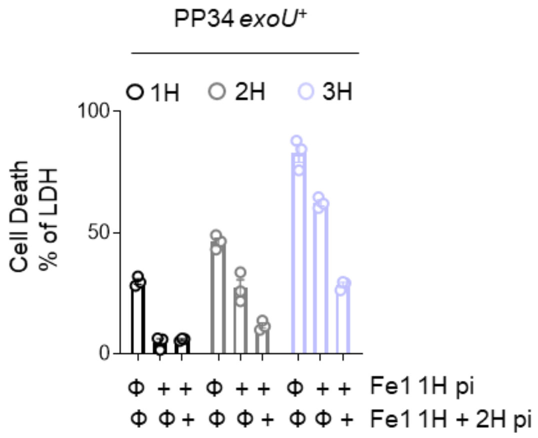

Measure of LDH release in Nlrc4-/- BMDMs infected with PP34 (MOI5) in presence of Ferrostatin-1 (Fe1, 10µM) for 3 hours. Each hour, fresh Ferrostatin-1 (10µM) was added to cells (+) or not (φ). “pi” refers to post-infection. Attension ici, tu n’as pas mis la ferro à 1h pré-infection et 1h post-infection, Faut voir avec Sali mais je crois que c’est 1h pré-infection puis ajouté ou non en même temps que l’infection.

Part III – Minor Issues: Editorial and Data Presentation Modifications

Please use this section for editorial suggestions as well as relatively minor modifications of existing data that would enhance clarity.

Reviewer #1: Line 88: a space is missing “balancein”

Line 168: and extra space is present “E xoU”

Line 236: Figure S3C is mentioned before S3B.

Line 262-264 is an incomplete sentence.

Line 325: a space is missing “toevaluate” and the word “rigger” is missing a “t” at the beginning. Furthermore, I recommend starting a new paragraph here as the authors begin describing an entirely different model system.

Line 332: This final sentence of the results is contradictory to earlier claims, “Altogether, our results identified lipid peroxidation as a pathological mechanism induced by the P. aeruginosa ExoU phospholipase both in mice and in human bronchial organoids.”. Earlier, authors make the careful point, concluded through experimentation, that ExoU does not induce lipid peroxidation, but rather exploits existing baseline lipid peroxidation to execute necrosis. This sentence undercuts that message, unless the authors are suggesting that ExoU induces lipid peroxidation in vivo but not in vitro. If that is the case, there does not seem to be specific evidence

for this.

Figure S3F is confusing, the labeling says CuOOH but the legend says ferrostatin-1. From the text it seems the error occurred in the legend, as ferrostatin-1 was not involved in this experiment.

According to reviewer suggestions, we have directly modified the text.

Reviewer #2:

Minor points:

1) Discuss whether the other patatin family proteins play a role in the absence of ExoU?

We have integrated some discussion and observation around mammalian patatin-like phaospholipases, including iPLA2beta and iPLA2gamma.

"Ferroptosis is thought to be a constitutively activated form of cell death that is kept under control through the activity of endogenous regulators of lipid peroxidation such as GPX4, FSP1-mediated coQ10 production, α-tocopherol (vitamin E). In addition, the host cellular calcium (Ca2+)-independent PLA2γ, the peroriredoxin Prdx6 PLA2 or the PLA2G6 (Ca2+-independent PLA2β) can cleave and remove preferentially peroxidised phospholipids, hence contributing to phospholipid peroxide detoxification [61,62,67–71]. It is important to notice that both the iPLA2beta and iPLA2g belong to the patatin-like phospholipase family, as ExoU, which suggests that this family of phospholipases might have some conserved affinities to peroxidzed phospholipids [72]. The activity of those phospholipases is tightly regulated by various cellular systems (e.g. ROS levels, calcium fluxes, phospholipid composition) that ensure an optimal but not dysregulated phospholipid cleavage [71]. To this regard, our findings that cellular phospholipid peroxidation is a strong enhancer of ExoU-induced pathological necrosis appears in first view counter intuitive. In this context, we envision that, as a virulence factor, ExoU activity does not follow host regulation and uses host peroxided phospholipids to boost its patatin-like A2 phospholipase activity allowing to aberrantly target and cleave host (peroxidised) phospholipids."

2) Fig S2(C) What does the asterisk represents?

The asterisk represents non-specific recognition by the antibody. This has been precised in the figure legend.

3) Does ExoU has a role in regulating transferrin uptake by the host cell?

We apologize, this is something we have not checked yet but that will be done in the frame of our future experiments.

Reviewer #3: 1. There are some typing mistakes, and the manuscript should be carefully revised.

According to reviewer suggestions, we have carefully proof read the entire manuscript and edited or modified typing mistakes.

2. The legend of Figure 2E describes a graphic with the time course measure of plasma membrane permeabilization using propidium iodide incorporation in Nlrc4-/- BMDMs infected with PP34, PP34exoU- or PP34exoUS142A in presence of Ferrostatin-1 (Fe1, 20µM). However, the figure shows only non-infected BMDM as well as PP34- and PP34exoUS142A-infected cells but no data from exoU- infected cells.

We thank reviewer 3 for pointing this mistake. This has been corrected directly in the figure legend.

PLOS authors have the option to publish the peer review history of their article (what does this mean?). If published, this will include your full peer review and any attached files.

If you choose "no", your identity will remain anonymous but your review may still be made public.

Do you want your identity to be public for this peer review? For information about this choice, including consent withdrawal, please see our Privacy Policy.

Reviewer #1: No

Reviewer #2: No

Reviewer #3: No

Figure Files:

While revising your submission, please upload your figure files to the Preflight Analysis and Conversion Engine (PACE) digital diagnostic tool, https://pacev2.apexcovantage.com. PACE helps ensure that figures meet PLOS requirements. To use PACE, you must first register as a user. Then, login and navigate to the UPLOAD tab, where you will find detailed instructions on how to use the tool. If you encounter any issues or have any questions when using PACE, please email us at figures@plos.org.

Data Requirements:

Please note that, as a condition of publication, PLOS' data policy requires that you make available all data used to draw the conclusions outlined in your manuscript. Data must be deposited in an appropriate repository, included within the body of the manuscript, or uploaded as supporting information. This includes all numerical values that were used to generate graphs, histograms etc.. For an example see here on PLOS Biology: http://www.plosbiology.org/article/info%3Adoi%2F10.1371%2Fjournal.pbio.1001908#s5.

The numerical values as well as original membranes have been included in a supporting file entitled "Data S1. Original immunoblotting membranes in the current study." and "Data S2. Numerical values obtained in the current study."

Reproducibility:

To enhance the reproducibility of your results, we recommend that you deposit your laboratory protocols in protocols.io, where a protocol can be assigned its own identifier (DOI) such that it can be cited independently in the future. Additionally, PLOS ONE offers an option to publish peer-reviewed clinical study protocols. Read more information on sharing protocols at https://plos.org/protocols?utm_medium=editorial-email&utm_source=authorletters&utm_campaign=protocols
